# Multi-omics Data Reveal the Effect of Sodium Butyrate on Gene Expression and Protein Modification in *Streptomyces*

**DOI:** 10.1016/j.gpb.2022.09.002

**Published:** 2022-09-15

**Authors:** Jiazhen Zheng, Yue Li, Ning Liu, Jihui Zhang, Shuangjiang Liu, Huarong Tan

**Affiliations:** 1 State Key Laboratory of Microbial Resources, Institute of Microbiology, Chinese Academy of Sciences, Beijing 100101, China; 2 College of Life Sciences, University of Chinese Academy of Sciences, Beijing 100049, China; 3 State Key Laboratory of Microbial Biotechnology, Shandong University, Qingdao 266237, China

**Keywords:** Silent gene cluster, *Streptomyces*, Sodium butyrate, Protein modification, Acetylome

## Abstract

Streptomycetes possess numerous gene clusters and the potential to produce a large amount of natural products. Histone deacetylase (HDAC) inhibitors play an important role in the regulation of histone modifications in fungi, but their roles in prokaryotes remain poorly understood. Here, we investigated the global effects of the HDAC inhibitor, **sodium butyrate** (SB), on marine-derived ***Streptomyces****olivaceus* FXJ 8.021, particularly focusing on the activation of secondary metabolite biosynthesis. The antiSMASH analysis revealed 33 secondary metabolite biosynthetic gene clusters (BGCs) in strain FXJ 8.021, among which the silent lobophorin BGC was activated by SB. Transcriptomic data showed that the expression of genes involved in lobophorin biosynthesis (*ge00097–ge00139*) and CoA-ester formation (*e.g.*, *ge02824*), as well as the glycolysis/gluconeogenesis pathway (*e.g*., *ge01661*), was significantly up-regulated in the presence of SB. Intracellular CoA-ester analysis confirmed that SB triggered the biosynthesis of CoA-ester, thereby increasing the precursor supply for lobophorin biosynthesis. Further acetylomic analysis revealed that the acetylation levels on 218 sites of 190 proteins were up-regulated and those on 411 sites of 310 proteins were down-regulated. These acetylated proteins were particularly enriched in transcriptional and translational machinery components (*e.g*., elongation factor GE04399), and their correlations with the proteins involved in lobophorin biosynthesis were established by protein–protein interaction network analysis, suggesting that SB might function via a complex hierarchical regulation to activate the expression of lobophorin BGC. These findings provide solid evidence that acetylated proteins triggered by SB could affect the expression of genes involved in the biosynthesis of primary and secondary metabolites in prokaryotes.

## Introduction

Streptomycetes are the most abundant source of natural antibiotics, providing more than half of medically important antimicrobial and antitumor agents. With the increase in antibiotic resistance and infectious disease outbreaks, including the recent coronavirus disease 2019 (COVID-19) pandemics, novel bioactive compounds are urgently needed to cope with emerging pathogens. In recent years, marine-derived *Streptomyces* strains were found to have the potential to produce new bioactive natural products [1]. With the rapid development of bioinformatics, a vast number of antibiotic biosynthetic gene clusters (BGCs) have been discovered from *Streptomyces* species [2]. Therefore, the known antibiotics are only the tip of the iceberg. However, most of these BGCs remain silent under laboratory culture conditions, even though they could potentially produce diverse bioactive products.

Various general strategies for activating or enhancing the biosynthesis of silent antibiotics have been developed, such as ribosome engineering [3], genetic manipulation of global or pathway-specific regulatory genes [4], [5], heterologous expression of gene clusters [6], [7], clustered regularly interspaced short palindromic repeats (CRISPR)/CRISPR-associated protein 9 (CRISPR/Cas9)-mediated genome editing [8], high-throughput elicitor screens (HiTES) [9], and co-cultivation of microorganisms [10]. More importantly, due to the complex diversity of *Streptomyces* under different culture conditions, establishment of more efficient strategies to activate these silent BGCs is highly desirable for seeking novel natural products. Recently, epigenetic regulation has become promising for discovering secondary metabolites. For example, in eukaryotes, histone deacetylases (HDACs) and histone methyltransferases (HMTs) can convert chromatins from an acetylated form to a methylated form. The post-translational modifications (PTMs) of histone can trigger the production of multiple secondary metabolites via modulating histone acetylation by small-molecule inhibitors of HDACs [11], [12], [13], [14]. Although the presence of histone is not defined in prokaryotes, different types of nucleoid-associated proteins (NAPs) (‘histone-like’) are widely present in bacteria [15], [16]. Intriguingly, some deacetylases in bacteria contain the domain of HDACs in eukaryotes [17], [18], implying that HDAC inhibitors may have the potential to influence protein acetylation in bacteria. The question of whether these inhibitors could regulate the biosynthesis of secondary metabolites in prokaryotes, especially in *Streptomyces,* is a hot issue worthy of study.

Here, we demonstrated that the silent lobophorin BGC in *Streptomyces olivaceus* FXJ 8.021 was activated by the addition of HDAC inhibitor, sodium butyrate (SB). Multi-omics and intracellular CoA-ester analyses revealed the complex cellular response of *S. olivaceus* FXJ 8.021 to SB, including the regulation of genes involved in lobophorin biosynthesis, glycolysis/gluconeogenesis, and carbon metabolism at the transcriptional, translational, and protein-acetylation levels. Our findings not only provide an efficient strategy for activating silent BGCs, but also deepen the understanding of the global effects of protein acetylation on primary and secondary metabolism in *Streptomyces*.

## Results

### Genomic features of *S. olivaceus* FXJ 8.021

*S. olivaceus* FXJ 8.021, a marine-derived strain, was isolated from the southwest Indian ridge-derived sediment at a depth of 3500 m. Its complete genome was sequenced on the single-molecule real-time sequencing (SMRT) platform of Oxford Nanopore Technologies (ONT). The complete genome consisting of a linear chromosome and a plasmid is 8,336,230 bp in length with an average GC content of 72.39%. The genome was predicted to encode 7385 proteins, 18 rRNAs, 66 tRNAs, and 63 non-coding RNAs (ncRNAs) (Table S1).

Genomic analysis of *S. olivaceus* FXJ 8.021 using antiSMASH 5.0 [19] predicted 33 secondary metabolite BGCs (Table S2). Among them, 14 BGCs were predicted to be involved in the biosynthesis of polyketide-derived secondary metabolites, including polyketide compounds, non-ribosome-peptide-derived secondary metabolites, and polyketide synthase–nonribosomal peptide synthase (PKS–NRPS)-derived hybrid metabolites. Additionally, 19 BGCs were predicted to be  responsible for the biosynthesis of terpenes, indoles, siderophores, or other categories. Notably, 14 out of the 33 predicted secondary metabolite BGCs exhibited less than 50% similarity to BGCs with known or characterized products, indicating that *S. olivaceus* FXJ 8.021 has the potential to produce novel natural products.

### SB activates the expression of silent lobophorin BGC

**Figure 1:**
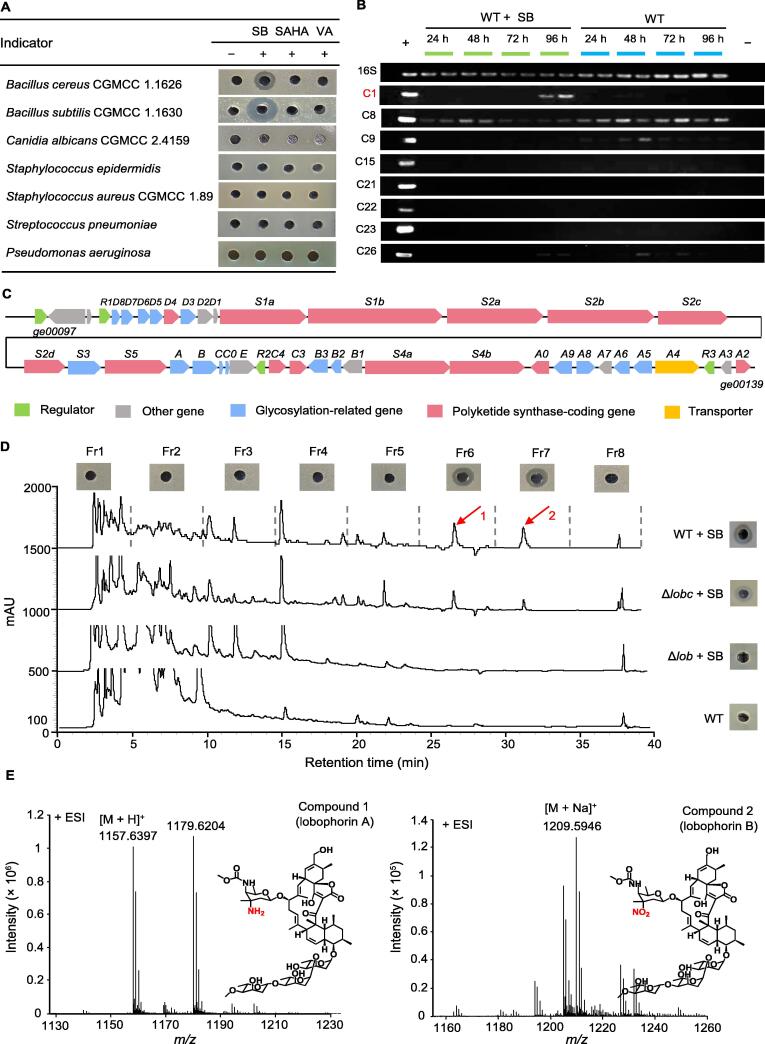
**Activation, identification, and characterization of the silent gene clusters mediated by HDAC inhibitors** **A.** Antimicrobial detection of fermentation broth of *S. olivaceus* FXJ 8.021 in ISP2 medium with or without HDAC inhibitors. + indicates ISP2 medium with HDAC inhibitors (SB, SAHA, and VA), and − indicates ISP2 medium without inhibitors (control). **B.** RT-PCR analysis of the core genes of seletcted BGCs in *S. olivaceus* FXJ 8.021 (WT). The genome DNA of *S. olivaceus* FXJ 8.021 was used as a positive (+) control, and distilled H_2_O served as a negative (−) control in RT-PCR. A subset of tested core genes from C1 (*ge00109*), C8 (*ge01054*), C9 (*ge01163*), C15 (*ge03905*), C21 (*ge05559*), C22 (*ge05622*), C23 (*ge05700*), and C26 (*ge06020*), are shown. The constitutive 16S rDNA-coding gene was used as an internal control in RT-PCR. **C.** Genetic organization of C1 in *S. olivaceus* FXJ 8.021. **D.** HPLC analysis and antimicrobial assessment of the fermentation broth of WT, Δ*lob*, and Δ*lobc* strains. A total of eight fractions were collected by continuous sampling based on time phases (fr1, 0–5 min; fr2, 5–10 min; fr3, 10–15 min; fr4, 15–20 min; fr5, 20–25 min; fr6, 25–30 min; fr7, 30–35 min; fr8, 35–40 min). The red arrows indicate compounds 1 and 2, and *B. subtilis* was used as the indicator strain. **E.** Structural identification of compound 1 and compound 2 by HRESI-MS. HDAC, histone deacetylase; ISP2, international *Streptomyces* project No. 2; SB, sodium butyrate; SAHA, suberoylanilide hydroxamic acid; VA, valproic acid; BGC, biosynthetic gene cluster; WT, wild-type; RT-PCR, reverse transcription polymerase chain reaction; HPLC, high-performance liquid chromatography; HRESI-MS, high-resolution electrospray ionization mass spectrometry; CGMCC, China General Microbiological Culture Collection Center; ESI, electron spray ionization.

HDAC inhibitors are widely used in the study of eukaryotic gene expression regulated by histone acetylation, including numerous classes of chemicals, such as short-chain fatty acids (SCFAs), hydroxyamate, and depsipeptides [16], [20]. Whether HDAC inhibitors could affect the biosynthesis and production of secondary metabolites in *Streptomyces* is a very interesting scientific question worthy of exploration. Here, the effects of SB, suberoylanilide hydroxamic acid (SAHA), and valproic acid (VA) on the biosynthesis of secondary metabolites in *S. olivaceus* FXJ 8.021 were investigated. Obvious inhibition zones against Gram-positive bacteria *Bacillus cereus* China General Microbiological Culture Collection Center (CGMCC) 1.1626 and *Bacillus subtilis* CGMCC 1.1630 were observed in the fermentation culture of *S. olivaceus* FXJ 8.021 upon the addition of SB, implying the production of bioactive compound(s) (Figure 1A).

To determine the gene clusters associated with the bioactive compound(s) in *S. olivaceus* FXJ 8.021, the transcriptional levels of the core genes in selected secondary metabolite BGCs were analyzed by reverse transcription polymerase chain reaction (RT-PCR). The result showed that a core gene from cluster 1 (C1) was transcribed in *S. olivaceus* FXJ 8.021 grown in the international *Streptomyces* project No. 2 (ISP2) medium supplemented with SB at 96 h, but not in the bacteria grown in ISP2 medium alone, revealing that C1 may be responsible for the biosynthesis of the bioactive compounds (Figure 1B). Furthermore, the antiSMASH analysis indicated that C1 has a high identity with the lobophorin BGC from *S. olivaceus* SCSIO T05 [21]. C1 consists of 43 open reading frames (*ge00097*–*ge00139*) (Figure 1C), and the predicted functions of proteins encoded by these genes are shown in Table S3. To validate whether C1 is directly responsible for the biosynthesis of bioactive compounds, the gene *ge00109*, encoding a unit of keto-synthase, was inactivated by in-frame deletion to generate the disruption mutant Δ*lob*. Both high-performance liquid chromatography (HPLC) analysis and antibacterial detection of fermentation broth showed that Δ*lob* strain completely lost its ability to produce active compounds, while its complementary strain (Δ*lobc*) restored the production of the active compounds in the presence of SB (Figure 1D). These results indicate that C1 is likely responsible for the biosynthesis of bioactive compounds.

To elucidate the chemical structures of these compounds, the fermentation broth of *S. olivaceus* FXJ 8.021 in the presence of SB was centrifuged and extracted with ethyl acetate. The extract was analyzed and fractionated by HPLC. A total of eight fractions (fr1, 0–5 min; fr2, 5–10 min; fr3, 10–15 min; fr4, 15–20 min; fr5, 20–25 min; fr6, 25–30 min; fr7, 30–35 min; fr8, 35–40 min) were collected. Fr6 and fr7 exhibited inhibitory activity against *B. subtilis* CGMCC 1.1630 (Figure 1D). Compounds 1 and 2 from fr6 and fr7 were respectively collected and then analyzed by high-resolution electrospray ionization mass spectrometry (HRESI-MS) and nuclear magnetic resonance (NMR). The [M+H]^+^ of compound 1 was *m/z* 1157.6397 on HRESI-MS (lobophorin A, C_61_H_92_N_2_O_19_, exact mass: 1156.6294), and the [M+Na]^+^ of compound 2 was *m/z* 1209.5946 (lobophorin B, C_61_H_90_N_2_O_21_, exact mass: 1186.6036) (Figure 1E). The ^1^H and ^13^C NMR data of compound 2 (Table S4; Figure S1) matched those of lobophorin B [22]. Taken together, compound 1 and compound 2 were determined to be the members of lobophorin.

### RNA sequencing analysis reveals differentially expressed genes triggered by SB

**Figure 2:**
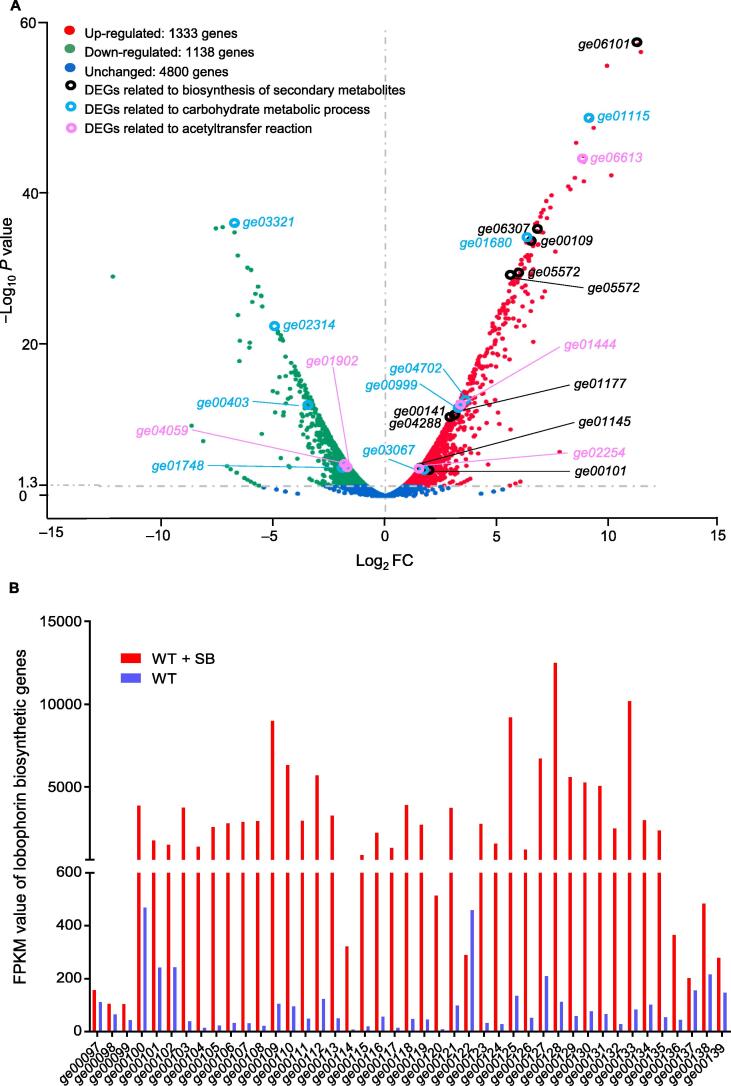
**RNA-seq analysis of *S. olivaceus* FXJ 8.021 in the presence or absence of SB** **A.** Volcano plot of DEGs in *S. olivaceus* FXJ 8.021 with the statistically significant difference (*P* < 0.05 and |log_2_ FC| > 1). Red, green, and blue colors indicate up-regulated, down-regulated, and unchanged genes, respectively. DEGs related to the GO categories in the following three dimensions are highlighted: biosynthesis of secondary metabolites (black color), carbohydrate metabolic process (blue color), and acetyltransfer reaction (pink color). **B.** FPKM values of DEGs situated in the lobophorin BGC of *S. olivaceus* FXJ 8.021 (WT + SB *vs.* WT). RNA-seq, RNA sequencing; DEG, differentially expressed gene; FC, fold change; GO, Gene Ontology; FPKM, fragments per kilobase of exon model per million reads mapped.

To understand the physiological changes of *Streptomyces* in the presence of SB, transcriptomic analysis of *S. olivaceus* FXJ 8.021 was performed. Since the production of lobophorin was observed on day 5, the cultures on day 4 and day 5 of fermentation were sampled for RNA sequencing (RNA-seq) analysis. The results showed that the transcriptional levels of 2471 genes (equivalent to 33% of all genes) were significantly affected by the addition of SB in fermentation broth on day 5 (|log_2_ fold change (FC)| > 1, *P* < 0.05) (Figure 2A; Table S5). Among the differentially expressed genes (DEGs), the expression of 1333 transcripts was up-regulated (shown in red), while the expression of 1138 transcripts was down-regulated (shown in green) after fermentation with the addition of SB. The log_2_ FC values of the expression of the up-regulated genes varied from 1 to 11.3, and those of the down-regulated genes ranged from −12.2 to −1. To validate the RNA-seq results, six DEGs were randomly selected for reverse transcription quantitative polymerase chain reaction (RT-qPCR) analysis. These included two up-regulated genes encoding a regulatory protein (*ge00100*, log_2_ FC = 3.0) and a beta-ketoacyl synthase (*ge00109*, log_2_ FC = 6.4), two down-regulated genes encoding a phytoene synthase (*ge00505*, log_2_ FC = −4.9) and a beta-ketoacyl synthase (*ge07198*, log_2_ FC = −2.6), and two unchanged genes encoding a methylisoborneol synthase (*ge00211*, log_2_ FC = 0.9) and an AMP-binding protein (*ge01054*, log_2_ FC = 0.2) (Figure S2). The results were consistent with those of RNA-seq analysis, indicating the reliability and accuracy of RNA-seq data.

To identify the significantly enriched biological processes regulated by SB, Gene Ontology (GO) and Kyoto Encyclopedia of Genes and Genomes (KEGG) pathway enrichment analyses were performed with the clusterProfiler R package [23], and *P* < 0.05 was considered significant. The results of GO analysis showed that up-regulated DEGs were highly enriched in carbohydrate metabolic process, cofactor binding, and transporter activity, while down-regulated DEGs were highly enriched in the metabolic process (Figure S3). In the KEGG pathway enrichment analysis, up-regulated DEGs were highly enriched in amino acid metabolism, oxidative phosphorylation, and starch and sucrose metabolism pathways, while down-regulated DEGs were highly enriched in microbial metabolism in carbon metabolism, diverse environments, and biosynthesis of cofactors (Figure S4). As shown in Figure 2A, DEGs related to the biosynthesis of secondary metabolites belong to the nagalamycin BGC (*e.g*., *ge06101*), lobophorin BGC (*e.g*., *ge00101*), butyrolactol A BGC (*e.g*., *ge01145*), friulimicin A BGC (*e.g*., *ge05572*), and hopene BGC (*e.g*., *ge06307*). Additionally, genes responsible for carbohydrate metabolic process (*ge01115*, *ge03321*, *ge01680*, *etc*.) and acetyltransfer reaction (*ge06613*, *ge04059*, *ge01444*, *etc*.) exhibited remarkable variations at the transcriptional level. Collectively, these findings suggest that SB globally alters the transcriptional levels of genes involved in the biosynthesis of metabolites in *Streptomyces.*

As expected, the transcriptomic data demonstrated that the expression of most lobophorin biosynthetic genes (*ge00097*–*ge00139*) was up-regulated in *S. olivaceus* FXJ 8.021 (WT + SB) compared with the control (WT) (Figure 2B; Table S6). The log_2_ FC values of the expression levels of the DEGs responsible for pentacyclic aglycon and trisaccharide chain formation were approximately 6.5, such as beta-ketoacyl synthase-coding genes (*lobS1a*, *lobS1b*, *lobS2b*, *lobS2c*, *lobS2d*, *etc*.) and glycosylation-related enzyme-coding genes (*lobD8*, *lobD7*, *lobD6*, *lobD5*, *lobD3*, *lobB1*, and *lobA8*). Moreover, the expression of *lobR1*, a positive regulatory gene in lobophorin biosynthesis, was also up-regulated (log_2_ FC = 3.0). Therefore, the overexpression of lobophorin BGC could contribute to the biosynthesis of lobophorin.

### SB globally modulates the carbohydrate metabolism in *S. olivaceus* FXJ 8.021

**Figure 3:**
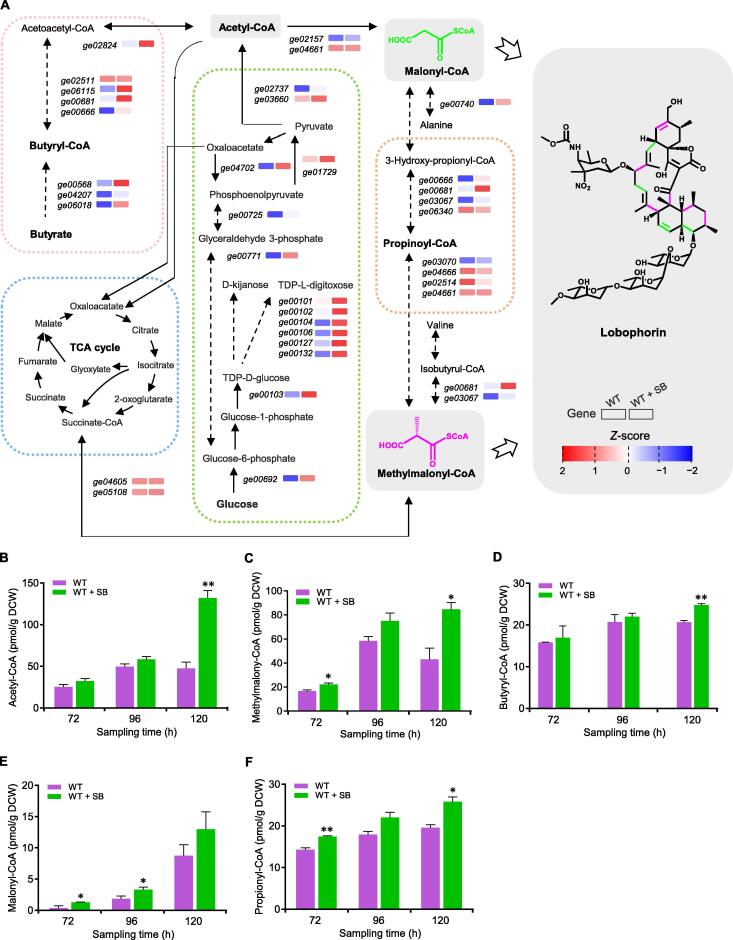
**Effect of SB on carbon metabolism in *S. olivaceus* FXJ 8.021** **A.** DEGs involved in carbon metabolism by RNA-seq analysis of *S. olivaceus* FXJ 8.021. The relative expression levels of DEGs in the strains without SB treatment (left) and with SB treatment (right) are indicated by small blocks. The relative expression levels were normalized by *z*-score standardization (blue to red indicates low to high). Dotted arrows indicate multiple-step reactions. The blue dotted frame indicates the TCA cycle, green for glycolysis, red for butyrate metabolism, and orange for propionate metabolism. Malonyl-CoA (chemical structure depicted in green) and methylmalonyl-CoA (chemical structure depicted in purple) are extender units for the biosynthesis of lobophorin. Intracellular concentrations of different CoA-esters in *S. olivaceus* FXJ 8.021 (WT) grown in ISP2 with or without SB are shown in the lower panels: acetyl-CoA (**B**), methylmalonyl-CoA (**C**), butyryl-CoA (**D**), malonyl-CoA (**E**), and propionyl-CoA (**F**). Student’s *t*-test was used to analyze the statistically significant differences (*, *P* < 0.05; **, *P* < 0.01). DCW, dry cell weight; TCA, tricarboxylic acid.

Based on current knowledge, the biosynthesis of lobophorin is initiated by a linear type I polyketide backbone assembled by 4 malonyl-CoA and 6 methylmalonyl-CoA, incorporated with a glycerol-derived three-carbon unit, which then undergoes an intricate cycloaddition reaction to form aglycone, equipped with a trisaccharide chain comprising three L-digitoxoses and a D-kejanose [24]. As a SCFA, SB could be converted to butyryl-CoA to produce ketone bodies and acetyl-CoA entering into the Krebs cycle [25], [26]. Hence, it is reasonable to assume that SB might affect the carbohydrate metabolism and the CoA precursor reservoir for lobophorin biosynthesis. Our further in-depth analysis revealed that the expression of DEGs related to CoA-ester biosynthesis increased significantly (log_2_ FC > 1; *P* < 0.05), implying the accumulation of lobophorin precursors (Figure 3A; Table S7). For example, the expression of most genes involved in the three main pathways for synthesizing methylmalonyl-CoA (one of the lobophorin precursors) was up-regulated: (1) genes (*ge04605* and *ge05108*) encoding enzymes responsible for isomerization of succinyl-CoA, (2) genes (*ge03070*, *ge04666*, *ge02514*, and *ge04661*) encoding enzymes responsible for carboxylation of propionyl-CoA, and (3) genes (*ge00681* and *ge03067*) encoding enzymes responsible for the conversion of isobutyryl-CoA to methylmalonyl-CoA by multi-step oxidation. Among these genes, the log_2_ FC values of the expression levels of *ge00681* and *ge03067* were approximately 2.5. Regarding the metabolic flux from acetyl-CoA to malonyl-CoA (another lobophorin precursor), the expression of acetyl-CoA carboxylase-coding genes (*ge04661* and *ge02157*) was not significantly changed. However, the expression of upstream genes (*ge00692*, *ge00771*, *ge00725*, *ge01729*, and *ge00740*) encoding enzymes involved in gluconeogenesis/glycolysis and alanine catabolism showed significant up-regulation (log_2_ FC ranging from 1.6 to 3.5), which could facilitate the accumulation of acetyl-CoA and malonyl-CoA. The expression of *ge04702*, which encodes the enzyme for the conversion of oxaloacetate to phosphoenolpyruvate related to the tricarboxylic acid (TCA) cycle and glycolytic pathway, was also up-regulated (log_2_ FC = 4.4). In addition, the expression of genes (*ge06018*, *ge00568*, *ge06115*, and *ge02824*) encoding enzymes responsible for the conversion of acetyl-CoA from pyruvate and butyrate was also up-regulated (log_2_ FC ranging from 1.6 to 2.8). As mentioned in Figure 2B, the expression of genes (*ge00101*, *ge00102*, *ge00103*, *ge00104*, *ge00106*, *ge00127*, and *ge00132*) encoding enzymes responsible for the formation of L-digitoxose and D-kijanose of lobophorin from TDP-D-glucose showed up-regulation. Additionally, the expression of *ge00692* encoding an enzyme responsible for the conversion of glucose-1-phosphate from glucose also increased (log_2_ FC = 1.9).

Considering that the expression of DEGs involved in the lobophorin biosynthetic pathway and carbon metabolism was up-regulated, it is interesting to evaluate the concentration changes of intracellular CoA-esters which are crucial precursors of TCA and lobophorin biosynthesis. We focused on five CoA-esters related to lobophorin biosynthesis and butyrate metabolism, including acetyl-CoA, malonyl-CoA, propionyl-CoA, methylmalonyl-CoA, and butyryl-CoA. At 120 h after culture, the concentrations of the intracellular acetyl-CoA [∼ 132 pmol/g dry cell weight (DCW), 1.8-fold increase] and methylmalonyl-CoA (∼ 85 pmol/g DCM, 1.0-fold increase) significantly increased in the ISP2 medium supplemented with SB compared with those in the ISP2 medium alone, and the concentrations of malonyl-CoA, butyryl-CoA, and propionyl-CoA showed a tendency of slight increase as well (Figure 3B–F). In summary, changes in the intracellular CoA-ester concentrations were consistent with the transcriptional variations of the relevant DEGs, demonstrating that the enhanced metabolic precursors induced by SB might facilitate the activation of lobophorin BGC.

### SB globally alters the protein acetylation level

**Figure 4:**
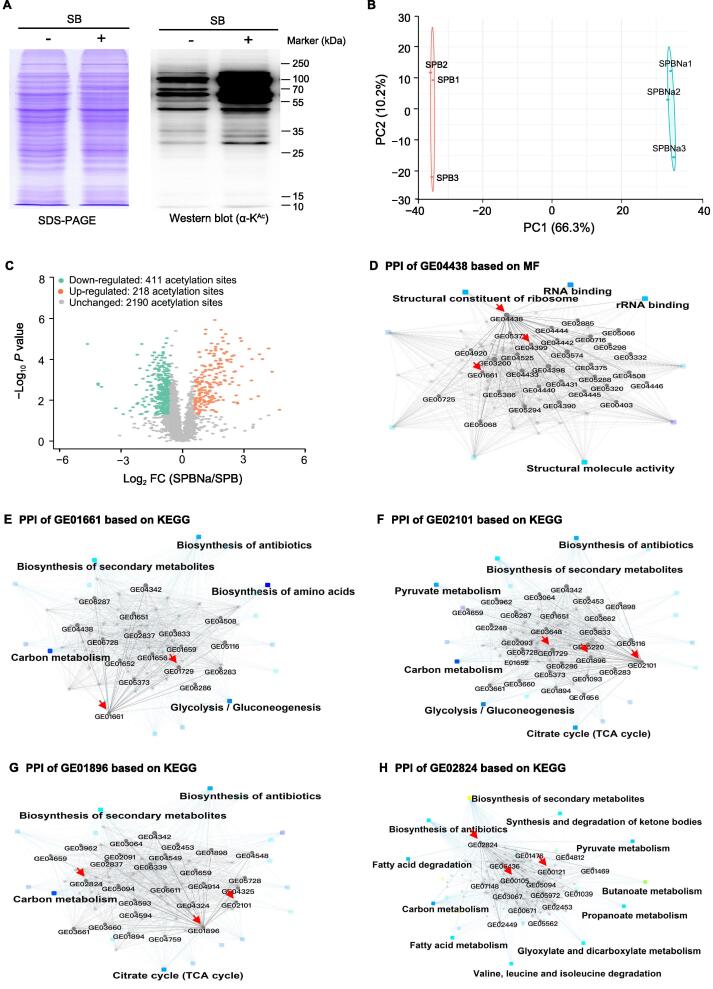
**SB-triggered acetylation changes of proteins involved in primary and secondary metabolism** **A.** SDS–PAGE and Western blotting analyses showing the acetylation profiles of the total proteins from *S. olivaceus* FXJ 8.021 with or without SB treatment. + indicates ISP2 medium with SB, and − indicates ISP2 medium without SB as control. **B.** The statistical analysis of acetylome profiling by PCA. **C.** Volcano plot showing the changes in acetylated peptide intensities of *S. olivaceus* FXJ 8.021 grown in ISP2 medium with or without SB (*P* < 0.05 and |log_2_ FC| > 0.6). Specifically, NALs of acetylated peptides with FC > 1.50 were considered up-regulated significantly (brown dots), and those with FC < 0.67 were considered down-regulated significantly (cyan dots) based on the recommended strict cutoff criteria [32]. **D.** PPI network of GE04438 was constructed based on MF. **E.**–**H.** PPI networks of GE01661 (E), GE02101 (F), GE01896 (G), and GE02824 (H) based on KEGG. The proteins mentioned in the main text are marked with red arrows. SDS–PAGE, sodium dodecyl sulfate–polyacrylamide gel electrophoresis; PCA, principal component analysis; NAL, normalized acetylation level; PPI, protein–protein interaction; MF, molecular function; KEGG, Kyoto Encyclopedia of Genes and Genomes; SPBNa, *Streptomyces* protein bio-analysis with SB; SPB, *Streptomyces* protein bio-analysis.

It has been reported that HDAC inhibitors can increase the acetylation level of both histone and non-histone proteins [*e.g*., transcription factor p53 and nuclear factor kappa B (NF-κB)], and modulate gene expression in eukaryotes [27], [28], [29], [30]. Furthermore, SB is a non-selective inhibitor of most HDACs except for HDAC-6 and HDAC-10 [31]. To evaluate the effect of SB on protein acetylation, sodium dodecyl sulfate–polyacrylamide gel electrophoresis (SDS–PAGE) and Western blotting analyses were performed to compare protein acetylation levels in *S. olivaceus* FXJ 8.021 with or without SB treatment. Since acetylation often occurs on lysine, an anti-acetyllysine antibody was selected to detect acetylation changes. Equal amounts of proteins were loaded and visualized in Coomassie brilliant blue staining gel (Figure 4A, left), and Western blotting analysis showed different acetylation degrees of proteins extracted from the two groups with or without SB (Figure 4A, right). Furthermore, high-resolution liquid chromatography with tandem mass spectrometry (LC–MS/MS) analysis was preformed, followed by quantitative proteomic and acetylomic analyses. *Streptomyces* protein bio-analysis with SB (SPBNa) indicates the experiment group, while *Streptomyces* protein bio-analysis (SPB) indicates the control group without SB. Principal component analysis (PCA) showed clear separation of distinct features occurring in SPBNa and SPB, with features of the same group clustered together, verifying the validity and reliability of the data (Figure 4B). As a result, 1473 proteins were identified to be acetylated with false discovery rate (FDR) < 1%. The relative intensities of the acetylated peptides were normalized against the changes in protein abundance owing to variations in protein expression to avoid possible biases of acetylation level [32], [33]. Finally, acetylated peptides with normalized acetylation levels (NALs) were used for acetylation dynamic analysis. Based on the recommended strict cutoff criteria [32], the ratios of acetylated peptides with NALs between SPBNa and SPB were calculated, and the mean value of the ratios from three replicates was used as the final FC. In brief, the acetylated peptides with FC > 1.50 or < 0.67 and *P* < 0.05 were considered significantly changed. Finally, 218 up-regulated acetylation sites and 411 down-regulated acetylation sites were identified after SB treatment (Figure 4C; Table S8). GO and KEGG pathway enrichment analyses showed that differentially acetylated proteins were enriched in transcriptional and translational machinery components (TTMCs), biosynthesis of antibiotics, gluconeogenesis/glycolysis pathway, and carbon metabolism (Figure S5; Table S8). How these changes in acetylation influence the primary and secondary metabolism of *S. olivaceus* FXJ 8.021 needs further studies.

### Acetylomic analysis reveals the relationship between acetylated TTMCs and lobophorin biosynthesis

Considering that differential acetylation levels of proteins and multiple acetylated sites might play important roles in regulating the biosynthesis of lobophorin, protein–protein interaction (PPI) networks of all the differentially acetylated proteins were constructed based on molecular function (MF) or KEGG by the STRING database and OmicsBean online software.

Lysine acetylation of ribosomal protein and elongation factor (EF) is critical for translational efficiency. For example, an acetylated variant of EF-Tu increased its translation efficiency in *Escherichia coli* [34], [35]. Here, we took EF-Tu GE04399 and 30S ribosomal protein GE04438 in *S. olivaceus* FXJ 8.021 as examples to elucidate the acetylation changes at different sites. Among the 12 acetylation sites in GE04399, 3 sites showed a significant increase in NALs (FC = 18.4, 18.4, and 2.3, respectively), and 1 site showed a slight decrease in NALs (FC = 0.6). Similarly, the NALs of 3 acetylation sites in 30S ribosomal protein GE04438 also exhibited significant changes (Table S8). These TTMCs showed significant direct and indirect interactions (GE04399/GE04438–GE01661/GE01729/GE02101/GE01896–GE02824–GE00105/GE00121) with proteins involved in gluconeogenesis/glycolysis and CoA-ester metabolism (Figure 4D–H), such as GE01661 (glyceraldehyde-3-phosphate dehydrogenase) (Figure 4E), GE01729 (pyruvate kinase), GE02101 (pyruvate dehydrogenase E1 component) (Figure 4F), GE01896 (dihydrolipoamide acetyltransferase component of pyruvate dehydrogenase) (Figure 4G), and GE02824 (acetyl-CoA acetyltransferase) (Figure 4H). Particularly, GE02824 plays a crucial role in multiple KEGG pathways, such as glycolysis gluconeogenesis, carbon metabolism, citrate cycle, fatty acid metabolism, butanoate metabolism, propanoate metabolism, valine degradation, and biosynthesis of antibiotics (GE00105 and GE00121 in the lobophorin BGC). These findings imply that the acetylated TTMCs might function at higher levels in the hierarchy of gene transcriptional regulation via acetylation changes, ultimately leading to the production of the silent lobophorin.

## Discussion

Dozens of secondary metabolic gene clusters can be found usually in a genome of *Streptomyces*, but only a few of them are expressed or have detectable products [36]. Since various factors can influence the regulatory network of *Streptomyces*, supplementation of small molecules might be a simple and effective strategy to activate silent BGCs. In this study, the roles of three HDAC inhibitors (SB, SAHA, and VA) were investigated in the activation of silent BGCs. Furthermore, the effect of SB on the global protein acetylation and activation of lobophorin BGCs was elucidated by multi-omics analysis.

PTMs are crucial for the diverse functions of various proteins. For example, methylation in rRNA structure was related to the dysregulation of protein synthesis [37]; succinylation of EF-Tu in *B. subtilis* could decrease the translational activity [38]; the mimic acetylation variant of EF-G in *E. coli* reduced the translational elongation rate [34], while an acetylated variant of EF-Tu increased the translational efficiency [35]. In this study, multi-omics analysis was firstly conducted to elucidate a full-scale landscape of the cellular response of *S. olivaceus* FXJ 8.021 to SB. We identified 49 acetylation sites on 5 EFs [EF-4 (GE02284), EF-G (GE04398), EF-P (GE01220), EF-Ts (GE05321), and EF-Tu (GE04399)]. Significantly up-regulated NALs of K3 and K5 in GE04399 (FC = 18.4 for both), as well as significantly down-regulated NALs of K394 in GE04399 (FC = 0.6) and K260 in GE04398 (FC = 0.5), were observed in SPBNa. In addition, several ribosome proteins (GE05320, GE04389, GE04437, *etc*.) also exhibited significant changes in acetylation levels. Different acetylation sites in the same protein may influence different functions in the corresponding pathway. Previous studies have reported that certain acetylated forms of EF lead to faster delivery of amino-acyl-tRNA to the ribosome, consequently resulting in an increased elongation rate [34], [35]. N-terminal acetylation of some proteins has been shown to enhance their stability [39], suggesting that similar effects might also occur on some TTMCs in *S. olivaceus* FXJ 8.021.

NAPs function as a global regulator in prokaryotes, as histone does in eukaryotes. DNA topoisomerases (DNA relaxation and chromosome decompaction), IHF (nucleoid compaction), Lsr2, and H-NS-like proteins facilitate the maintenance of the compact and dynamic nucleoid [40]. In this work, DNA topoisomerases (GE05478, GE05506, and GE03958) showed significant changes at the acetylation level. For example, the NAL of K548 in GE05506 was significantly down-regulated in SPBNa (FC = 0.4), while that of K7 was up-regulated (FC = 2.5). However, their relationship with lobophorin production remains elusive. Regarding NAPs GE03425 and GE03860, we found these two proteins repressed the biosynthesis of lobophorin, and the disruption of both *ge03425* and *ge03860* led to the production of a novel lobophorin derivative (unpublished data). Protein quantification showed that GE03425 did not exhibit significant changes in protein abundance, while the expression of GE03860 showed up-regulation based on the proteomic data. We speculated that the acetylation of GE03425 and GE03860 might be altered with the addition of SB, leading to the biosynthesis of lobophorin. We therefore attempted to analyze their acetylation levels. Unfortunately, normalized acetylation quantification data for GE03425 and GE03860 were not obtained from the acetylome profiling. One possible reason might be the relatively low abundance or instability of the acetylated GE03425 and GE03860, posing challenges in the enrichment of lysine-acetylated peptides for analysis. Another possibility is that the sampling time of fermentation for acetylomic analysis might not be optimal for capturing sufficient amounts of acetylated GE03425 and GE03860. Considering the global influence of SB on protein acetylation and gene transcription in *Streptomyces*, further investigation into its roles in modifying the conserved NAPs in bacteria is worthwhile in the future.

In eukaryotes, lysine acetylation serves as a kind of fine-tuning mechanism of protein activity and is a reversible reaction catalyzed by acetyltransferase and deacetylase [41], [42]. Although the HDAC inhibitor SB induces protein hyper-acetylation by altering the activity of HDACs, not all HDACs are inhibited by SB, such as HDAC-6 and HDAC-10 (class II) [16], [31]. In *S. olivaceus* FXJ 8.021, only three deacetylases contain HDAC domains. Among them, GE00002 and GE07072 contain a sirtuin catalytic domain (showing a high similarity with HDAC class III), and GE04099 possesses another typical HDAC domain different from sirtuin. Furthermore, deacetylases in *Streptomyces* are versatile in the PTMs of proteins, indicating the complex regulatory network mediated by HDACs and their inhibitors. Therefore, clarification of their roles would provide significant insights into the cross-regulation of primary and secondary metabolism in prokaryotes.

## Conclusion

**Figure 5:**
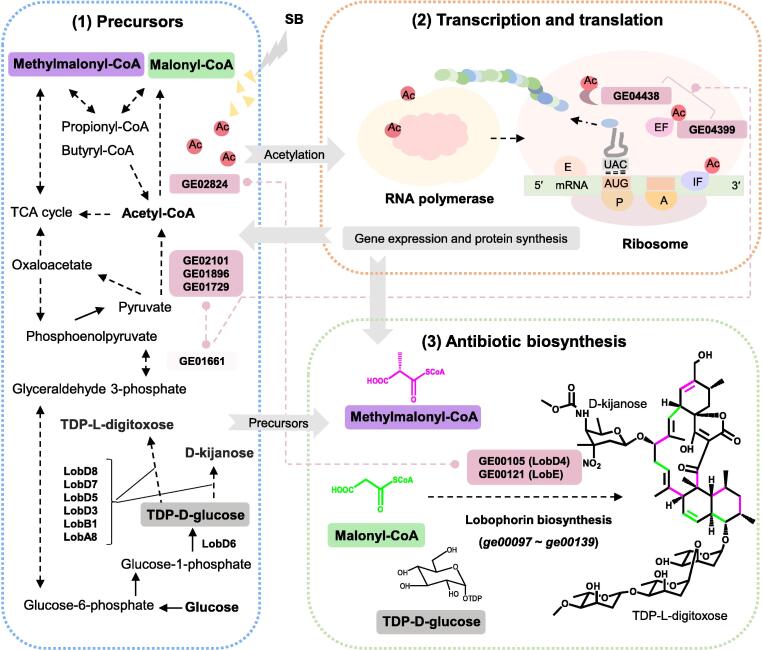
**Cellular response of *S. olivaceus* FXJ 8.021 to SB** A proposed model for the expression of a silent lobophorin BGC activated by the HDAC inhibitor SB in *Streptomyces* spp. Dotted arrows represent multiple-step reactions. Dashed lines with bidirectional dots (rose-red color) indicate PPIs. The metabolic pathways of precursors for lobophorin are indicated by a blue dotted frame, the transcriptional and translational machinery system is indicated by an orange dotted frame, and the biosynthesis of lobophorin is indicated by a green dotted frame. In general, the activation mechanism can be ascribed to two aspects: (1) SB treatment results in the accumulation of methylmalonyl-CoA, malonyl-CoA, D-kijanose, and TDP-L-digitoxose; and (2) CoA-esters exceeding the consumption capacity of the central metabolic pathway would trigger extensive protein acetylation, which could, in turn, regulate the expression of multiple genes related to lobophorin biosynthesis.

In this study, we demonstrated that HDAC inhibitor SB could activate the silent lobophorin BGC in *Streptomyces*. Based on the results of multi-omics analyses as well as the concentration changes of intracellular CoA-esters, we proposed a model for the activation of the silent lobophorin BGC by SB (Figure 5). It is suggested that SB leads to the accumulation of the intermediate precursors of lobophorin (*e.g*., methylmalonyl-CoA and malonyl-CoA), which directly promotes the production of lobophorin. On the other hand, the accumulated CoA-esters can be utilized for PTMs, *e.g*., acetyl-CoA (a donor of an acetyl group for protein acetylation). The addition of SB also triggers the acetylation of TTMCs (*e.g*., GE04438 having 11 acetylation sites and GE04399 having 12 acetylation sites), which in turn globally influences the transcription and translation of genes encoding enzymes involved in glycolysis/gluconeogenesis, CoA-ester formation, and lobophorin biosynthesis. Taken together, SB exerts its function by globally affecting the protein acetylation and enhancing precursor supply to activate lobophorin biosynthesis.

## Materials and methods

### Strains, plasmids, primers, and growth conditions

All strains and plasmids used in this study are listed in Table S9, and primers are listed in Table S10. *S. olivaceus* FXJ 8.021 and their derivatives were cultivated at 28 °C on mannitol-soya (MS) solid medium containing 20 g/l mannitol (Catalog No. M813423, Macklin, Shanghai, China), 20 g/l soya flour, and 20 g/l agar. For shaking flask fermentation, yeast extract-malt extract (YEME) liquid medium supplemented with 3 g/l yeast extract (Catalog No. Y110517, Aladdin, Shanghai, China), 5 g/l peptone (Catalog No. P6277, Macklin), 3 g/l malt extract (Catalog No. MK-M828184, Macklin), 200 g/l sucrose (Catalog No. S6216, Macklin), and 10 g/l glucose (Catalog No. D810594, Macklin) was used for seed culturing. ISP2 medium, containing 4 g/l glucose, 4 g/l yeast extract, and 10 g/l malt extract, was used for liquid fermentation. SB medium was ISP2 medium supplemented with 6.25 mM HDAC inhibitor SB (Catalog No. S102954, Aladdin) before fermentation. *E. coli* JM109 was used for plasmid construction, and *E. coli* ET12567/pUZ8002 was used for plasmid conjugation transfer from *E. coli* to *Streptomyces* as described previously [43]. Indicator strains, including *Bacillus cereus* CGMCC 1.1626, *Bacillus subtilis* CGMCC 1.1630, *Canidia albicans* CGMCC 2.4159, *Staphylococcus epidermidis*, *Staphylococcus aureus* CGMCC 1.89, *Streptococcus pneumoniae*, and *Pseudomonas aeruginosa*, were grown in Luria-Bertani broth (LB) medium containing 10 g/l tryptone (Catalog No. T6276, Macklin), 5 g/l yeast extract (Catalog No. Y110517, Aladdin), 10 g/l NaCl (Catalog No. C111533, Aladdin), and 10 g/l agar (Catalog No. A800728, Macklin) at 37 °C for antibacterial assessment. For the selection of *E. coli* transformants in LB, the final concentration of apramycin or kanamycin was 100 μg/ml. For *Streptomyces*, the final concentration of apramycin or nalidixic acid was 25 μg/ml.

### Genome sequencing, assembly, and gene annotation

High-quality genomic DNA was used to construct the library by the ligation sequencing kit (Catalog No. SQK-LSK109, ONT, Kidlington, UK) and then sequenced on the SMRT platform of ONT. Clean data were used to assemble the subreads into 2 scaffolds and generate a full length of 8.34 Mb using Canu [44]. The genome of *S. olivaceus* FXJ 8.021 contains a total of 7385 protein-coding genes. Moreover, the tRNAs, rRNAs, and other ncRNA-coding genes were predicted by tRNAscan-SE [45], Infernal 1.1 [46], and Rfam [46], respectively. The annotation of predicted genes was conducted by BLAST.

### Construction of *ge00109* disruption mutant and its complementary strain

To generate Δ*lob*, the plasmid pKC1139-*lobUD* was firstly constructed using pKC1139 vector in *S. olivaceus* FXJ 8.021. For the construction of pKC1139-*lobUD*, primer pairs LobUp-F/LobUp-R and LobDn-F/LobDn-R listed in Table S10 were used for PCR amplification of the upstream and downstream fragments of *ge00109* (PKS-coding gene in C1), respectively. The PCR products of lobUp and lobDn were digested with *Xba*I/*Hin*dIII and *Hin*dIII/*Eco*RI, and then purified and inserted into the *Xba*I/*Eco*RI site of pKC1139 to generate pKC1139-*lobUD*. After *E. coli–Streptomyces* conjugal transfer, the transformants were screened at 38 °C in the presence of apramycin, then at 28 °C in the absence of apramycin. Finally, Apr^S^ colonies were confirmed by PCR analysis using primers Lobyz-F/Lobyz-R to obtain the correct *ge00109* disruption mutant, named as Δ*lob*.

For the construction of the complementary strain Δ*lobc*, the *ge00109* gene was amplified using primers 00109-F1/R1 and 00109-F2/R2. Prior to PCR amplification, 00109-R1 and 00109-F2 were phosphorylated. The PCR products digested with *Xba*I and *Eco*RI were purified and inserted into the corresponding sites of pSET152 to generate pSET152-109. The resulting pSET152*-*109 was further confirmed by DNA sequencing and then was introduced into Δ*lob* to obtain the complementary strain, which was designated as Δ*lobc*.

### Fermentation, extraction, and isolation

Spore suspensions of *S. olivaceus* FXJ 8.021 were inoculated into YEME medium and fermented for 48 h at 28 °C with 220 r/min as the seed culture. Then, 5% of seed cultures were transferred into SB medium. After 10 days, the fermentation broth was extracted with an equal volume of ethyl acetate three times, and the resulting ethyl acetate solution was evaporated to dryness. The crude extract was separated by a semi-preparative HPLC system using a reverse-phase column (ZORBAX, C18, 5 μm, 9.4 mm × 250 mm) with ultraviolet (UV) detection at 254 nm and 280 nm on Agilent 1260 infinity series (Agilent technologies, Santa Clara, CA). For the HPLC program, 1 ‰ formic acid (Catalog No. F809712, Macklin) in water was used as solvent A; methanol (Catalog No. M116118, Aladdin) was used as solvent B. The gradient elution procedure was as follows: 5% to 100% B (linear gradient, 0–30 min), 100% B (30–35 min), 100% to 5% B (35–36 min), 5% B (36–40 min), with a flow rate of 2 ml/min. The desired fractions were further purified under the modified program: 89% B (0–25 min) with the same condition as mentioned above.

### Detection of bioactivity

Fermentation broth of *S. olivaceus* FXJ 8.021 and its derivatives were added into the wells of LB agar plate containing 1% indicator strain. The inhibition zones were observed after incubation for 18–24 h at 37 °C.

### Extraction and quantification of intracellular CoA-esters

Acetyl-CoA, malonyl-CoA, methylmalonyl-CoA, and butyryl-CoA were extracted according to the previous protocol [47]. Briefly, 0.2 g of powdered cells were transferred into a pre-cooled tube containing 1.5 ml of 10% ice-cold trichloroacetic acid (TCA) (Catalog No. T0699, Sigma Aldrich, Saint Louis, MO) followed by vigorous vortex at 0 °C for 3 min. The supernatant from TCA suspension was collected by centrifugation (12,000 *g* for 10 min at 0 °C). To remove TCA, the culture was extracted with 2 ml of pre-chilled diethyl ether (−20 °C). The aqueous phase was lyophilized, dissolved in 300 μl of pre-cooled buffer [25 mM ammonium formate (Catalog No. A100187, Aladdin), pH 4.6, 2% methanol], and analyzed by LC–MS/MS. The data were represented as the mean value of three independent replicates and analyzed by GraphPad Prism 5.0. Error bars indicate  standard deviation (SD). Student’s *t*-test was used to analyze the statistically significant differences (*, *P* < 0.05; **, *P* < 0.01).

### Analysis of transcriptomic data and RT-qPCR

Samples of *S. olivaceus* FXJ 8.021 grown in ISP2 medium or SB medium were collected on day 4 and day 5. RNA extraction was performed using RNAprep pure cell/bacteria kit (Catalog No. DP430, TIANGEN, Beijing, China) according to the manufacturer’s instructions. RNA quality was assessed by the RNA nano 6000 assay kit (Catalog No. 5067-1511, Agilent, Santa Clara, CA). The details of library construction, RNA-seq, quality control, read mapping, and differential expression analysis are shown in File S1. For the validation of RNA-seq data, RT-qPCR was performed as described previously [48]. The primers used for RT-qPCR analysis are listed in Table S10, and the 16S rDNA-coding gene was used as the internal control.

### Determination of protein acetylation levels in *S. olivaceus* FXJ 8.021

For the assessment of the protein acetylation levels, mycelia of *S. olivaceus* FXJ 8.021 fermented in ISP2 or SB medium were harvested. Equal amounts of total proteins (20 μg) were separated using 12% SDS–PAGE and stained with Coomassie brilliant blue R-250 (Catalog No. 04821616, MP biomedicals, Santa Ana, CA). The same amounts of proteins separated on another 12% SDS–PAGE were transferred onto the PVDF membranes (Catalog No. 1620264, BIO-RAD, Hercules, CA) for Western blotting analysis to detect acetylation levels. The blocking buffer consisted of 100 mM Tris-HCl (pH 7.5), 0.5% (v/v) Tween-20 (Catalog No. T6335, Macklin), and 1% peptone (Catalog No. BD211677, BD Biosciences, NJ). The primary and secondary antibody buffer contained 100 mM Tris-HCl (pH 7.5), 0.05% (v/v) Tween-20, and 0.1% peptone. Pan anti-acetyllysine antibody (Catalog No. PTM-101, PTM BIO, Hangzhou, China) was used as the primary antibody, and it was incubated with the PVDF membranes overnight at 4 °C. Chemiluminescent Western blot reagents (Catalog No. RPN2236, GE Healthcare, Chicago, IL) were used for visualization by an ImageQuant LAS 4000 mini (GE Healthcare).

### Acetylomic analysis of *S. olivaceus* FXJ 8.021

The *S. olivaceus* FXJ 8.021 was cultured in ISP2 or SB medium, and after fermentation, the mycelia were washed three times with cold phosphate buffer solution (PBS). Then, the collected mycelia were frozen and ground into fine powder in liquid nitrogen, and lysed in lysis buffer containing 8 M urea (Catalog No. CS5709, Sigma-Aldrich), 1% protease inhibitor cocktail, 3 μM trichostatin A (TSA; Catalog No. T129665, Aladdin), and 50 mM nicotinamide (NAM; Catalog No. N105042, Aladdin). Subsequently, the mycelia with lysis buffer were sonicated. After centrifugation at 4 °C, the proteins were precipitated using 10% of cold TCA for 2 h at −20 °C, washed with pre-cold ice acetone, and then dissolved in buffer I [8 M urea, 100 mM triethanolamine borate (Catalog No. T7408, Sigma-Aldrich), pH 8.0]. For protein digestion, the proteins were reduced with 5 mM dithiothreitol (Catalog No. 20290, Thermo Fisher Scientific Waltham, MA) for 30 min at 56 °C and alkylated with 11 mM iodoacetamide (Catalog No. I131590, Aladdin) for 15 min at room temperature in dark. Then, trypsin was added at an enzyme:substrate ratio of 1:50 for overnight digestion and 1:100 for a second 4-h digestion. The enrichment of lysine-acetylated peptides was performed using acetylation antibody beads (Catalog No. PTM-104, PTM BIO). The acetylated peptides were re-suspended in buffer A [0.1% formic acid (Catalog No. F809712, Macklin) and 2% acetonitrile (Catalog No. A104440, Aladdin)] and separated by the EASY-nLC 1200 ultra-high performance liquid chromatography (UPLC) system (Thermo Fisher scientific) with a home-made reverse-phase analytical column (25 cm length, 75 μm i.d.). More details about data processing and bioinformatic analysis are shown in File S1.

## Data availability

The genomic and transcriptomic data have been deposited in the Genome Sequence Archive [49] at the National Genomics Data Center, Beijing Institute of Genomics, Chinese Academy of Sciences / China National Center for Bioinformation (GSA: CRA007837), which are publicly accessible at https://ngdc.cncb.ac.cn/gsa/. The proteomic and acetylomic data have been deposited in the China National Microbiology Data Center (NMDC; BioProject: NMDC10018160), which are publicly accessible at https://nmdc.cn/resource/genomics/sample?keyword=NMDC10018160.

## Competing interests

The authors declare that they have no competing interests.

## CRediT authorship contribution statement

**Jiazhen Zheng:** Methodology, Investigation, Validation, Formal analysis, Writing – original draft, Writing – review & editing, Data curation. **Yue Li:** Methodology, Investigation, Funding acquisition, Writing – review & editing, Data curation. **Ning Liu:** Methodology, Investigation, Formal analysis, Software. **Jihui Zhang:** Methodology, Formal analysis, Funding acquisition, Writing – review & editing. **Shuangjiang Liu:** Conceptualization, Supervision, Resources, Writing – review & editing. **Huarong Tan:** Conceptualization, Supervision, Resources, Funding acquisition, Writing – review & editing. All authors have read and approved the final manuscript.

## Supplementary material

Supplementary File S1 **Detailed methods of transcriptomic and acetylome analyses**

Supplementary Figure S1 **NMR spectra of lobophorin B A.** The 1H-NMR spectrum of lobophorin B. **B.** The ^13^C NMR spectrum of lobophorin B. NMR, nuclear magnetic resonance.

Supplementary Figure S2 **RT-qPCR transcriptional analysis for the validation of RNA-seq results** Verification of DEGs by RT-qPCR, including beta-ketoacyl synthase-coding gene (*ge00109*, log2 FC = 6.4), transcriptional regulatory protein-coding gene (*ge00100*, log2 FC= 3.0), methylisoborneol synthase-coding gene (*ge00211*, log2 FC = 0.9), AMP-binding protein-coding gene (*ge01054*, log2 FC = 0.2), phytoene synthase-coding gene (*ge00505*, log2 FC = –4.9), and beta-ketoacyl synthase (*ge07198*, log2 FC = –2.6). Total RNAs were isolated from *S. olivaceus* FXJ 8.021 (WT) with or without the addition of SB. The constitutive expression of the 16S rDNA-coding gene was used as an internal control. The results are presented as mean + standard deviations (SDs) of three independent experiments. Student’s *t*-test was used to analyze the statistically significant difference (*, *P* < 0.05; **, *P* < 0.01). RT-qPCR, reverse transcription quantitative polymerase chain reaction; DEG, differentially expressed gene; FC, fold change; WT, wild type; SB, sodium butyrate; SD, standard deviation.

Supplementary Figure S3 **GO enrichment of DEGs in *S. olivaceus* FXJ 8.021 A.** GO enrichment of up-regulated genes. **B.** GO enrichment of down-regulated genes. The count is the total number of DEGs annotated to each GO term. The horizontal axis indicates the ratio of the number of DEGs annotated to a certain term (*P*_adj_ ≤ 0.05) to the total number of genes in *S. olivaceus* FXJ 8.021 genome assigned to that term. *P*_adj_, adjusted *P* value; GO, gene ontology.

Supplementary Figure S4 **KEGG enrichment of DEGs in S. olivaceus FXJ 8.021 A.** KEGG enrichment of up-regulated genes. **B.** KEGG enrichment of down-regulated genes. The count is the total number of DEGs annotated to each KEGG term. The horizontal axis indicates the ratio of the number of DEGs annotated to a certain term (*P*_adj_ ≤ 0.05) to the total number of genes in *S. olivaceus* FXJ 8.021 genome assigned to that term. KEGG, kyoto encyclopedia of genes and genomes.

Supplementary Figure S5 **PPI for MF and KEGG analyses of acetylated proteins A.** PPI based on MF. **B.** PPI based on KEGG. PPI network analysis was performed based on STRING database version 9.1 and OmicsBean online software. MF, molecular function; PPI, protein–protein interaction.

Supplementary Table S1 **Genomic features of *S. olivaceus* FXJ 8.021**

Supplementary Table S2 **antiSMASH analysis of BGC in *S. olivaceus* FXJ 8.021**

Supplementary Table S3 **Prediction of functions of proteins encoded by lobophorin biosynthetic genes**

Supplementary Table S4 **^1^H and ^13^C NMR assignment of lobophorin B (measured at 500 MHz in CDCl_3_)**

Supplementary Table S5 **Transcriptomic data of *S. olivaceus* FXJ 8.021 on day 5**

Supplementary Table S6 **Transcriptomic data of lobophorin BGC in *S. olivaceus* FXJ 8.021**

Supplementary Table S7 **Transcriptomic data of genes related to CoA-ester pathways in *S. olivaceus* FXJ 8.021**

Supplementary Table S8 **Acetylome profiling of *S. olivaceus* FXJ 8.021**

Supplementary Table S9 **Strains and plasmids used in this study**

Supplementary Table S10 **Primers used in this study**
