# Supplementary material for: Multi-omics Data Reveal the Effect of Sodium Butyrate on Gene Expression and Protein Modification in Streptomyces
Source: Genomics Proteomics Bioinformatics. 2022 Sep 15;21(6):1149–62. doi: 10.1016/j.gpb.2022.09.002 (PMC11082262; doi:10.1016/j.gpb.2022.09.002)
Supplement: Supplementary File S1 — Detailed methods of transcriptomic and acetylome analyses [file mmc1.docx]

**File S1 Detailed methods of transcriptomic and acetylome analyses**

**Transcriptomic analysis**

*Library construction and RNA-sequencing*

The TruSeq stranded mRNA library prep kit (Catalog No. 20020594, Illumina, San Diego, CA) was used to construct the libraries of complementary DNA according to the manufacturer’s instructions. To ensure the quality of RNA library, the library was firstly quantified by Qubit2.0 fluorometer (Thermo Scientific™, Waltham, MA), and the insert size of the library was detected by Agilent 2100 bioanalyzer (Agilent Technologies, Santa Clara, CA). Reverse transcription quantitative polymerase chain reaction (RT-qPCR) was used to accurately quantify the effective concentration of the library. Then the qualified libraries were sequenced by the Illumina NovaSeq 6000 (Illumina) using 150 bp read length.

*Quality control and reads mapping*

For obtaining the clean data, reads containing adapter, N base, and low-quality reads were removed from raw data. Moreover, Q20, Q30, and GC contents of clean data were calculated. The following analyses were performed based on the high-quality clean data. Reads were mapped to the *S. olivaceus* FXJ 8.021 genome sequence with Bowtie2 (2.3.4.3) [1].

*Differential expression analysis*

Differentially expressed genes between sodium butyrate treatment and the original medium were analyzed by the edgeR R package (3.24.3) [2]. The resulting *P* was adjusted using the Benjamini & Hochberg method for controlling the false discovery rate. *P* < 0.05 and |log_2_ fold change| > 1 were set as the threshold for significant differentially expressed genes. The genes or samples with similar expression patterns in the heat map will be gathered together. The color in each square did not reflect the gene expression value, but the value obtained after the homogenization of the expression data (*z*-score).

*Functional enrichment analysis*

Gene ontology (GO) and kyoto encyclopedia of genes and genomes (KEGG) enrichment were implemented by the clusterProfiler R package (3.8.1) [3]. *P* < 0.05 was considered significantly enriched by differentially expressed genes.

**Acetylome analysis in *S. olivaceus* FXJ 8.021**

*Enrichment of lysine acetylated peptides*

The tryptic peptides were re-dissolved in the sampling buffer, including 50 mM Tris-HCl, 100 mM NaCl, 1 mM EDTA (Catalog No. E809069, Macklin, Shanghai, China), and 0.5% NP-40 (Catalog No. N8030, Solarbio, Beijing, China, pH 8.0), then incubated with prewashed acetylation antibody beads (Catalog No. PTM-104, PTM Bio, Hangzhou, China) at 4 ℃ overnight with gentle shaking. Then the beads were washed four times with sampling buffer and twice with distilled water. Prior to high-performance liquid chromatography with tandem mass spectrometry (HPLC/MS/MS) analysis, the bound peptides were eluted with 0.1% trifluoroacetic acid, dried by SpeedVac, and cleaned with C18 ZipTips (Catalog No. Z719986, Millipore, Sigma Aldrich, MO).

*LC-MS/MS analysis*

The liquid phase conditions are as follows: 5% – 25% B (0.1% formic acid and 90% acetonitrile) over 60 min, 25% to 35% B for 22 min, 35% to 80% B for 4min, and 80% B for the last 4 min at a constant flow rate of 450 nL/min. The resulting peptides were analyzed by using a tandem mass spectrometry (MS/MS) in Q Exactive^TM^ HF-X (ThermoFisher Scientific) coupled online to ultra-high performance liquid chromatography (UPLC). Intact peptides were detected in the Orbitrap analyzer at a resolution of 60,000. The normalized collision energy was set to 28, and ion fragments were detected at a resolution of 30,000. The top 20 precursor ions from each MS scan were isolated, fragmented, and measured in the linear ion trap. Fixed the first mass was set as 100 m/z.

*Data processing and database searches*

The resulting MS/MS data were analyzed using MaxQuant version 1.6.15.0 [4]. Tandem mass spectra were searched in the UniProt database. Acetylation (N-terminal, Lys) and oxidation (Met) were included in the search as the variable modifications. Cysteine carbamidomethylation was set as a fixed modification. The false discovery rate was adjusted to < 1%.

*Functional enrichment analysis*

GO annotation of proteome was performed based on the UniProt-GOA database (<http://www.ebi.ac.uk/GOA/>). The InterPro database was used to annotate protein functions based on the protein sequence alignment method [5]. KEGG database was used to annotate protein pathways using online service tools KAAS and KEGG mapper. *P* < 0.05 was considered significant.

**References**

[1] Langmead B, Salzberg SL. Fast gapped-read alignment with Bowtie 2. Nat Methods 2012;9:357–9.

[2] Chen Y, Lun ATL, Smyth GK. From reads to genes to pathways: differential expression analysis of RNA-Seq experiments using Rsubread and the edgeR quasi-likelihood pipeline. F1000Res 2016;5:1438.

[3] Yu G, Wang LG, Han Y, He QY. clusterProfiler: an R package for comparing biological themes among gene clusters. OMICS 2012;16:284–7.

[4] Jürgen C, Matthias M. MaxQuant enables high peptide identification rates, individualized p.p.b.-range mass accuracies and proteome-wide protein quantification. Nat Biotechnol 2008;26:1367–72.

[5] Jones P, Binns D, Chang HY, Fraser M, Li W, Mcanulla C, et al. InterProScan 5: genome-scale protein function classification. Bioinformatics 2014;30:1236–40.
