# Supplementary figures and images for: Multi-omics Data Reveal the Effect of Sodium Butyrate on Gene Expression and Protein Modification in Streptomyces
Source: Genomics Proteomics Bioinformatics. 2022 Sep 15;21(6):1149–62. doi: 10.1016/j.gpb.2022.09.002 (PMC11082262; doi:10.1016/j.gpb.2022.09.002)

**A**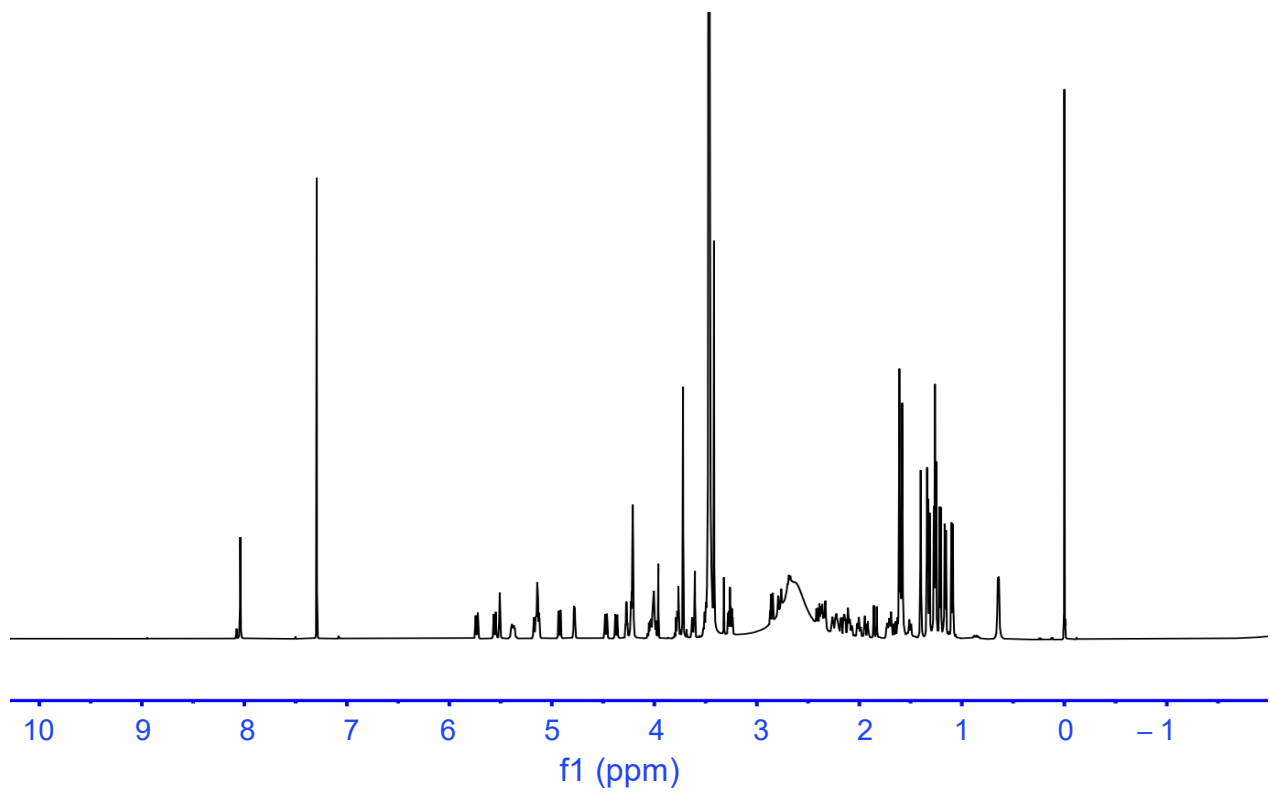**B**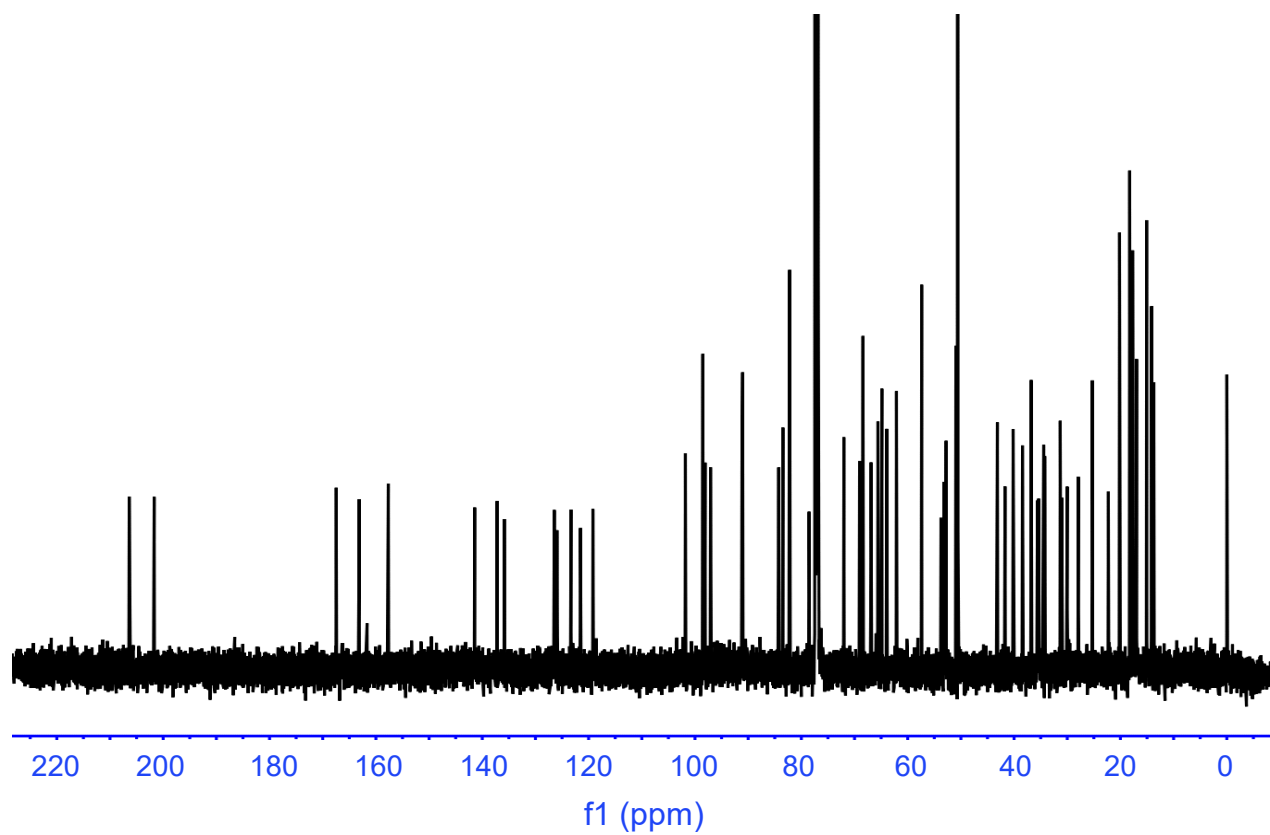

Supplement: Supplementary Figure S1 — NMR spectra of lobophorin B A. The 1H-NMR spectrum of lobophorin B. B. The 13C NMR spectrum of lobophorin B. NMR, nuclear magnetic resonance. [file mmc2.pdf]

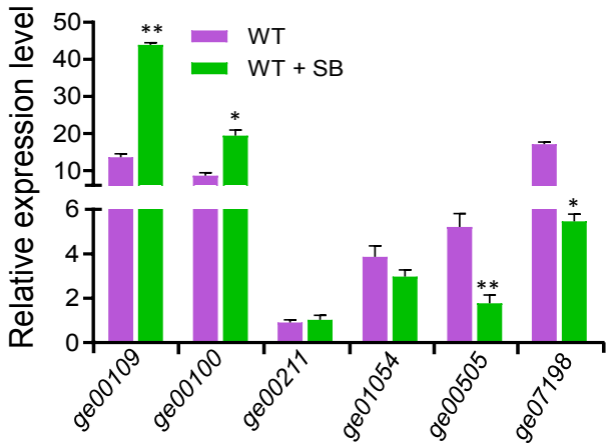

Supplement: Supplementary Figure S2 — RT-qPCR transcriptional analysis for the validation of RNA-seq results Verification of DEGs by RT-qPCR, including beta-ketoacyl synthase-coding gene (ge00109, log2 FC = 6.4), transcriptional regulatory protein-coding gene (ge00100, log2 FC= 3.0), methylisoborneol synthase-coding gene (ge00211, log2 FC = 0.9), AMP-binding protein-coding gene (ge01054, log2 FC = 0.2), phytoene synthase-coding gene (ge00505, log2 FC = –4.9), and beta-ketoacyl synthase (ge07198, log2 FC = –2.6). Total RNAs were isolated from S. olivaceus FXJ 8.021 (WT) with or without the addition of SB. The constitutive expression of the 16S rDNA-coding gene was used as an internal control. The results are presented as mean + standard deviations (SDs) of three independent experiments. Student’s t-test was used to analyze the statistically significant difference (*, P < 0.05; **, P < 0.01). RT-qPCR, reverse transcription quantitative polymerase chain reaction; DEG, differentially expressed gene; FC, fold change; WT, wild type; SB, sodium butyrate; SD, standard deviation. [file mmc3.pdf]

A

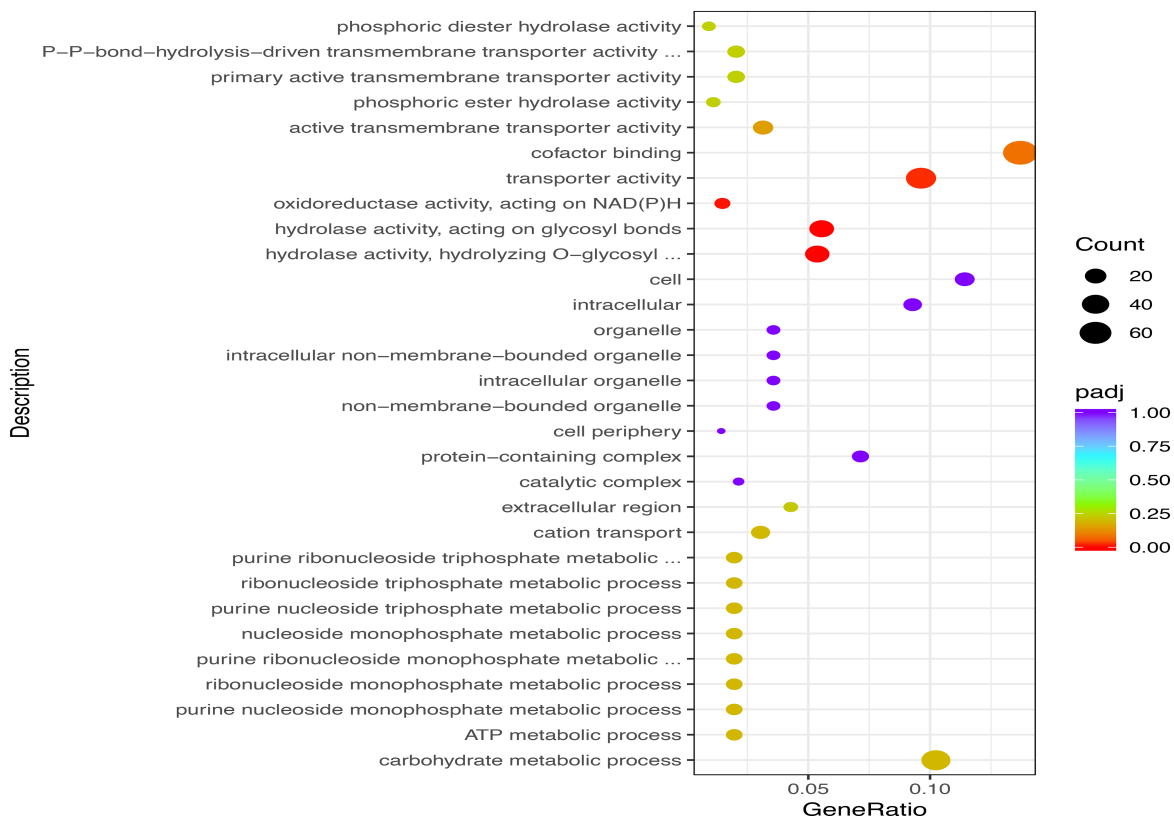

B

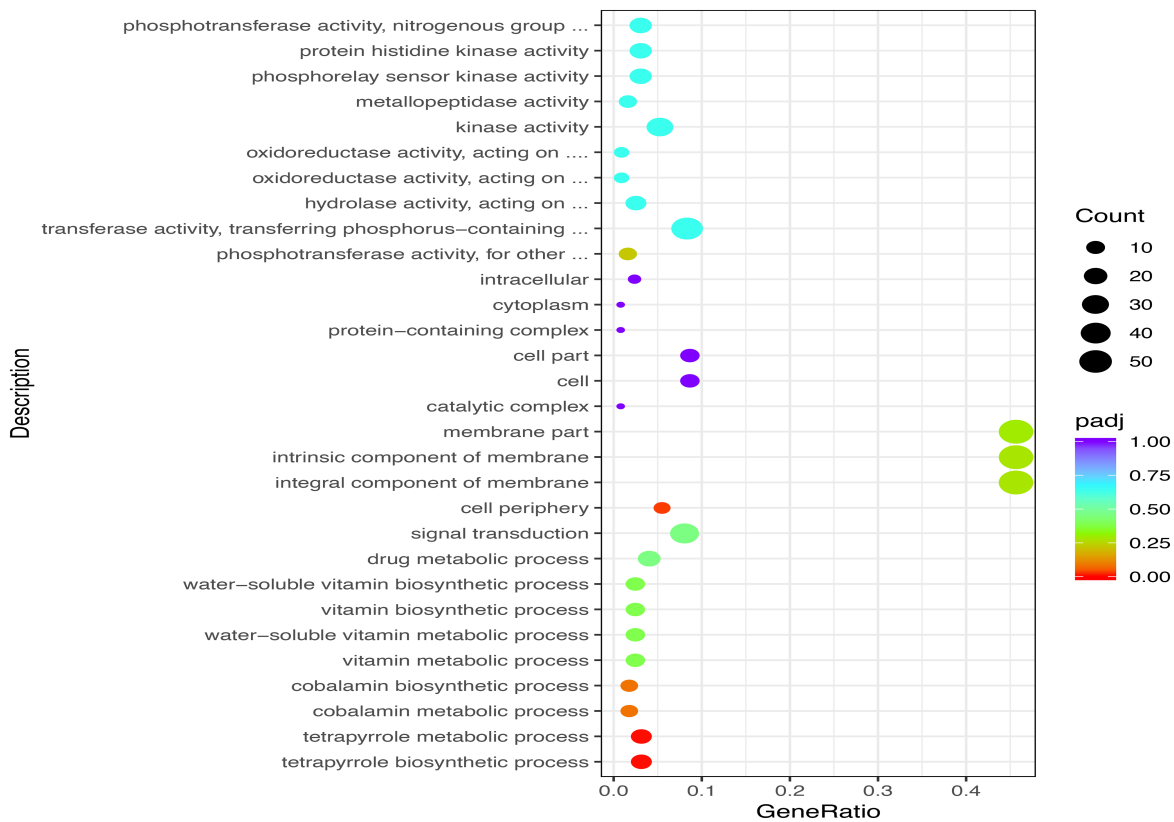

Supplement: Supplementary Figure S3 — GO enrichment of DEGs in S. olivaceus FXJ 8.021 A. GO enrichment of up-regulated genes. B. GO enrichment of down-regulated genes. The count is the total number of DEGs annotated to each GO term. The horizontal axis indicates the ratio of the number of DEGs annotated to a certain term (Padj ≤ 0.05) to the total number of genes in S. olivaceus FXJ 8.021 genome assigned to that term. Padj, adjusted P value; GO, gene ontology. [file mmc4.pdf]

A

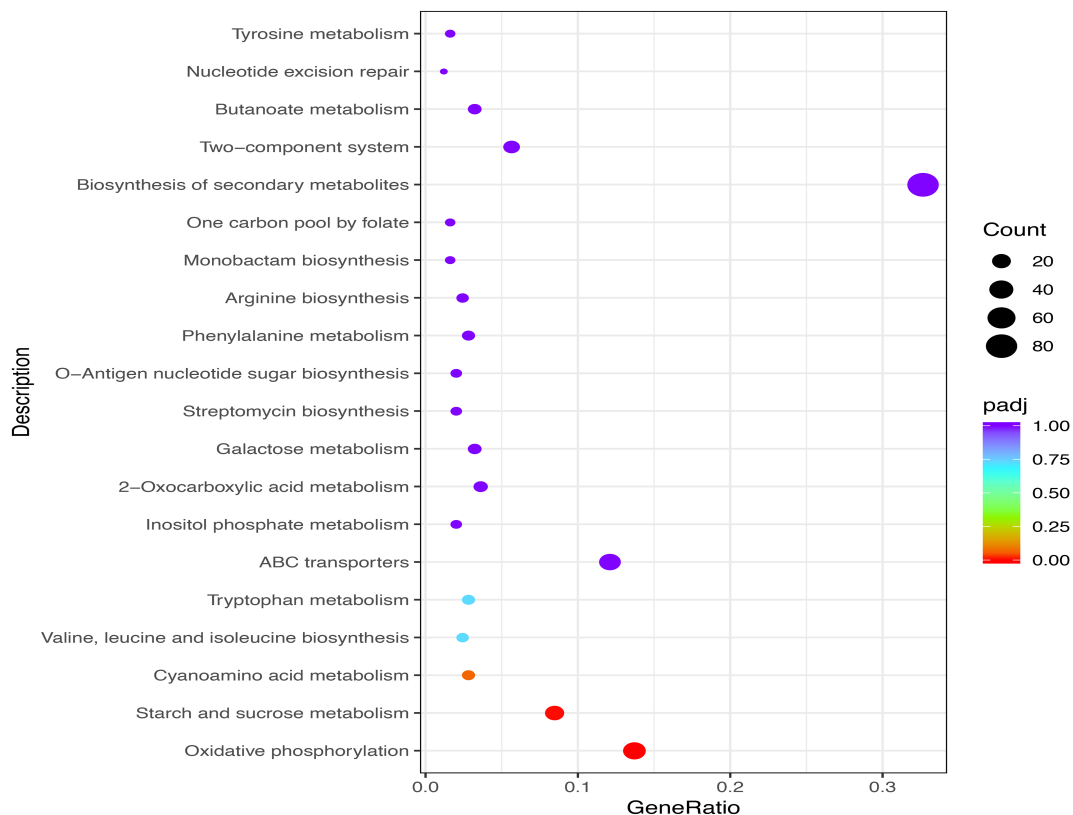

B

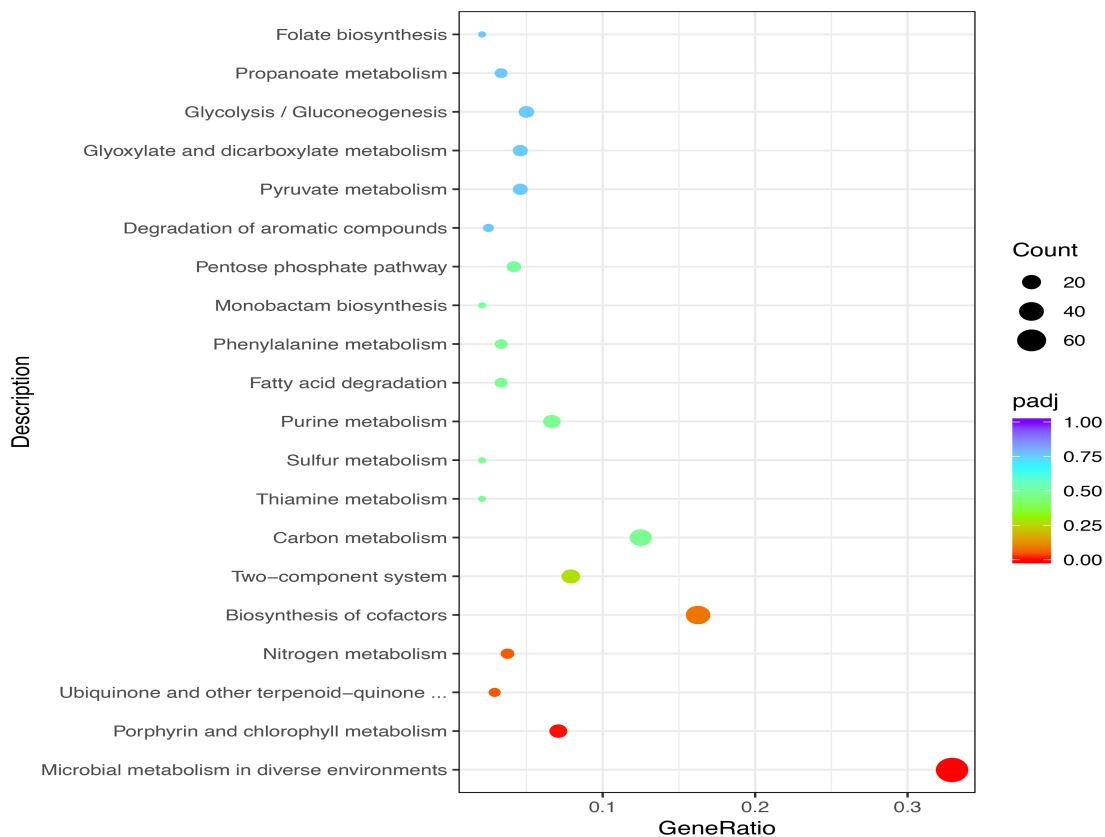

Supplement: Supplementary Figure S4 — KEGG enrichment of DEGs in S. olivaceus FXJ 8.021 A. KEGG enrichment of up-regulated genes. B. KEGG enrichment of down-regulated genes. The count is the total number of DEGs annotated to each KEGG term. The horizontal axis indicates the ratio of the number of DEGs annotated to a certain term (Padj ≤ 0.05) to the total number of genes in S. olivaceus FXJ 8.021 genome assigned to that term. KEGG, kyoto encyclopedia of genes and genomes. [file mmc5.pdf]

**A**

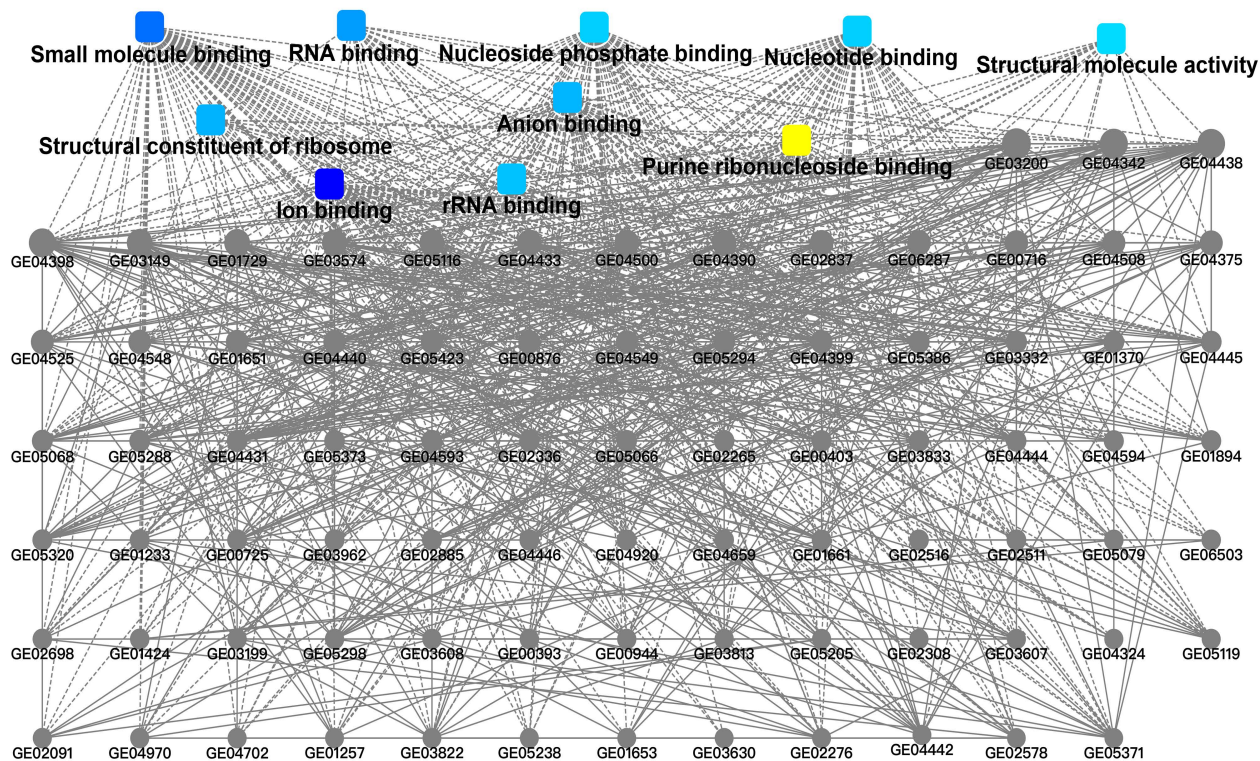

**B**

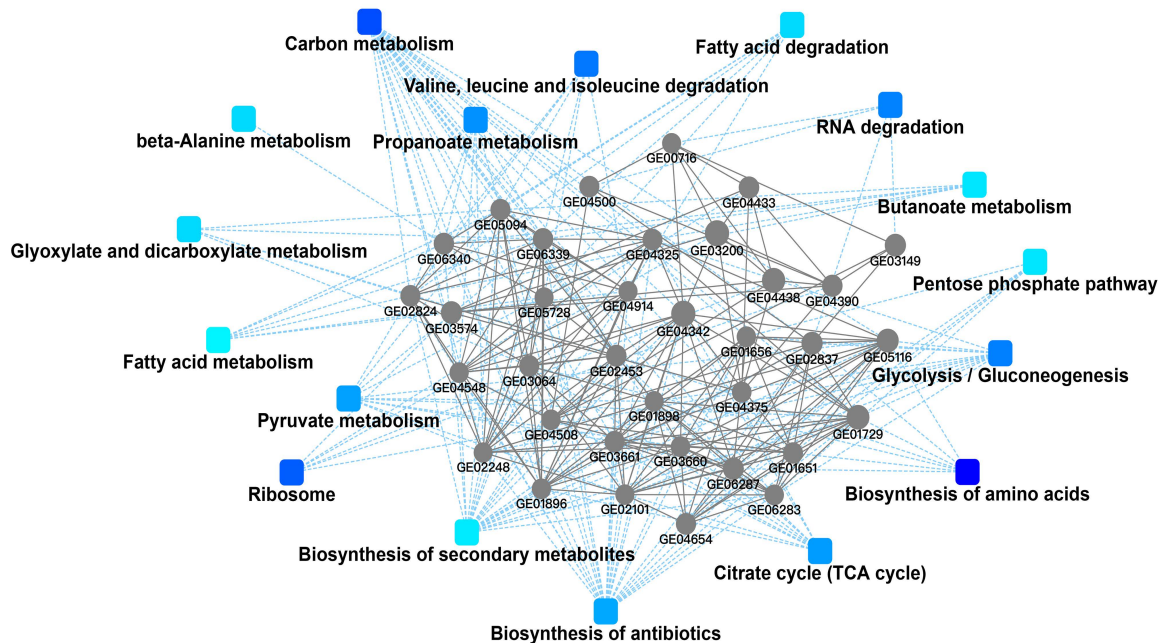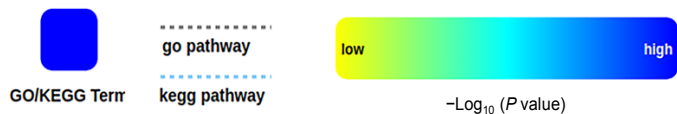

Supplement: Supplementary Figure S5 — PPI for MF and KEGG analyses of acetylated proteins A. PPI based on MF. B. PPI based on KEGG. PPI network analysis was performed based on STRING database version 9.1 and OmicsBean online software. MF, molecular function; PPI, protein–protein interaction. [file mmc6.pdf]
