# Supplementary material for: Multi-omics Data Reveal the Effect of Sodium Butyrate on Gene Expression and Protein Modification in Streptomyces
Source: Genomics Proteomics Bioinformatics. 2022 Sep 15;21(6):1149–62. doi: 10.1016/j.gpb.2022.09.002 (PMC11082262; doi:10.1016/j.gpb.2022.09.002)
Supplement: Supplementary Table S1 — Genomic features of S. olivaceus FXJ 8.021 [file mmc7.docx]

**Table S1 Genomic features of *S. olivaceus* FXJ 8.021**

| **Feature** | **Value** |
| --- | --- |
| Genome size (bp) | 8,336,230 |
| Average GC content (%) | 72.39 |
| Protein-coding genes | 7385 |
| Total size of protein-coding genes (bp) | 7,263,399 |
| rRNAs number | 18 |
| tRNAs number | 66 |
| Other non-coding RNA | 63 |
| Secondary metabolite gene clusters number | 33 |

*Note*: bp, base pair.
