# Supplementary material for: Multi-omics Data Reveal the Effect of Sodium Butyrate on Gene Expression and Protein Modification in Streptomyces
Source: Genomics Proteomics Bioinformatics. 2022 Sep 15;21(6):1149–62. doi: 10.1016/j.gpb.2022.09.002 (PMC11082262; doi:10.1016/j.gpb.2022.09.002)
Supplement: Supplementary Table S2 — antiSMASH analysis of BGC in S. olivaceus FXJ 8.021 [file mmc8.docx]

**Table S2 antiSMASH analysis of BGC in *S. olivaceus* FXJ 8.021**

| **BGC** | | **Type** | **Position** | | **Most similar known cluster** |
| --- | --- | --- | --- | --- | --- |
|  |  |  | **From** | **To** |  |
| Region 1.1 | T1PKS, NRPS, T3PKS | | 99,492 | 312,248 | Lobophorin A |
| Region 1.2 | Terpene | | 327,224 | 346,920 | Ebelactone |
| Region 1.3 | T3PKS | | 369,666 | 410,527 | Germicidin |
| Region 1.4 | Indole | | 559,424 | 580,551 | 5-isoprenylindole-3-carboxylate β-D-glycosyl ester |
| Region 1.5 | Terpene | | 631,923 | 655,546 | Carotenoid |
| Region 1.6 | Amglyccycl | | 833,765 | 855,003 | Β-D-galactosylvalidoxylamine-A |
| Region 1.7 | T3PKS | | 1,084,117 | 1,125,229 | Herboxidiene |
| Region 1.8 | NRPS | | 1,198,053 | 1,245,732 | Rimosamide |
| Region 1.9 | T1PKS | | 1,335,923 | 1,408,058 | Butyrolactol A |
| Region 1.10 | Ectoine | | 1,841,594 | 1,851,992 | Ectoine |
| Region 1.11 | Melanin | | 2,789,488 | 2,800,114 | Melanin |
| Region 1.12 | Lassopeptide | | 2,859,770 | 2,882,291 | Ssv-2083 |
| Region 1.13 | Siderophore | | 2,895,934 | 2,906,766 | Desferrioxamin B / desferrioxamine E |
| Region 1.14 | Thiopeptide | | 3,859,321 | 3,891,926 | Diazepinomicin |
| Region 1.15 | Lanthipeptide | | 4,404,472 | 4,432,659 | Sbi-06990 a1 / sbi-06989 a2 |
| Region 1.16 | NRPS | | 4,705,712 | 4,765,340 | Phosphonoglycans |
| Region 1.17 | Betalactone | | 5,459,664 | 5,485,229 | Julichrome Q3-3 / julichrome Q3-5 |
| Region 1.18 | Terpene | | 5,486,320 | 5,506,111 | Albaflavenone |
| Region 1.19 | T2PKS | | 5,556,696 | 5,629,292 | Spore pigment |
| Region 1.20 | Siderophore | | 6,086,801 | 6,097,036 |  |
| Region 1.21 | NRPS, T1PKS | | 6,183,495 | 6,294,770 | Friulimicin A / friulimicin B / friulimicin C / friulimicin D |
| Region 1.22 | NRPS | | 6,298,360 | 6,345,317 | Telomycin |
| Region 1.23 | NRPS, T1PKS | | 6,391,257 | 6,437,345 | Xiamycin A |
| Region 1.24 | Terpene | | 6,522,977 | 6,544,508 | Geosmin |
| Region 1.25 | Siderophore | | 6,692,145 | 6,705,317 |  |
| Region 1.26 | NRPS | | 6,741,319 | 6,786,942 | Diisonitrile antibiotic SF2768 |
| Region 1.27 | NRPS, nucleoside | | 6,823,879 | 6,861,645 | Nogalamycin |
| Region 1.28 | Terpene, NRPS | | 7,172,605 | 7,245,665 | Hopene |
| Region 1.29 | T1PKS, NRPS-like | | 7,300,365 | 7,375,528 | Divergolide A / divergolide B / divergolide C / divergolide D |
| Region 1.30 | Terpene | | 7,680,348 | 7,701,376 | Versipelostatin |
| Region 1.31 | Bacteriocin | | 7,710,546 | 7,720,761 | Informatipeptin |
| Region 1.32 | NRPS | | 7,923,355 | 7,974,280 | Coelichelin |
| Region 1.33 | T1PKS | | 8,112,476 | 8,201,052 | Elaiophylin |

*Note*: BGC, biosynthetic gene cluster; PKS, polyketide synthase; NRPS, nonribosomal peptide synthase; PKS-NRPS, polyketide synthase-nonribosomal peptide synthase.
