# Supplementary material for: Multi-omics Data Reveal the Effect of Sodium Butyrate on Gene Expression and Protein Modification in Streptomyces
Source: Genomics Proteomics Bioinformatics. 2022 Sep 15;21(6):1149–62. doi: 10.1016/j.gpb.2022.09.002 (PMC11082262; doi:10.1016/j.gpb.2022.09.002)
Supplement: Supplementary Table S3 — Prediction of functions of proteins encoded by lobophorin biosynthetic genes [file mmc9.docx]

**Table S3 Prediction of functions of proteins encoded by lobophorin biosynthetic genes**

| **Gene** | **Proposed function** | **Identity** | **Protein homologue** |
| --- | --- | --- | --- |
| *ge00097* | LysR family transcriptional Regulator | 98% | LuxR, AGI99509.1 |
| *ge00098* | Type I polyketide synthase | 50% | Crotonyl-CoA reductase, AGI99482.1 |
| *ge00099* | Putative protein | 100% | Putative protein, AGC09474.1 |
| *ge00100* | TetR type regulatory protein | 99% | LobR1, AGI99506.1 |
| *ge00101* | Sugar 5-epimerase | 99% | LobD8, AGI99505.1 |
| *ge00102* | Sugar 3-ketoreductase | 99% | LobD7, AGI99504.1 |
| *ge00103* | Sugar nucleotidyltransferase | 98% | LobD6, AGI99503.1 |
| *ge00104* | Sugar 4,6-dehydratase | 99% | LobD5, AGI99502.1 |
| *ge00105* | FAD-dependent oxidoreductase | 100% | LobD4, AGI99501.1 |
| *ge00106* | Sugar 3-aminotransferase | 98% | LobD3, AGI99500.1 |
| *ge00107* | Sugar 3-C-methyl transferase | 98% | LobD2, AGI99499.1 |
| *ge00108* | Unknown | 98% | LobD1, AGI99498.1 |
| *ge00109* | Type I polyketide synthase | 97% | LobS1a, AGI99497.1 |
| *ge00110* | Type I polyketide synthase | 96% | LobS1b, AGI99497.1 |
| *ge00111* | Type I polyketide synthase | 94% | LobS2a, AGI99496.1 |
| *ge00112* | Type I polyketide synthase | 97% | LobS2b, AGI99496.1 |
| *ge00113* | Type I polyketide synthase | 97% | LobS2c, AGI99496.1 |
| *ge00114* | Type I polyketide synthase | 84% | LobS2d, AGI99496.1 |
| *ge00115* | Type I polyketide synthase | 95% | LobS3, AGI99495.1 |
| *ge00116* | Type I polyketide synthase | 97% | LobS5, AGI99494.1 |
| *ge00117* | FAD-dependent oxidoreductase | 98% | LobA, AGI99493.1 |
| *ge00118* | Ketoacyl acylcarrier protein synthase III | 99% | LobB, AGI99492.1 |
| *ge00119* | FkbH-like protein | 98% | LobC, AGI99491.1 |
| *ge00120* | ACP | 99% | LobC0, AGI99490.1 |
| *ge00121* | Acyltransferase-like protein | 97% | LobE, AGI99489.1 |
| *ge00122* | TetR type regulatory protein | 98% | LobR2, AGI99488.1 |
| *ge00123* | Glycosyltransferase | 99% | LobC4, AGI99487.1 |
| *ge00124* | Glycosyltransferase | 99% | LobC3, AGI99486.1 |
| *ge00125* | FAD-dependent oxidoreductase | 96% | LobB3, AGI99485.1 |
| *ge00126* | Thioesterase | 99% | LobB2, AGI99484.1 |
| *ge00127* | Sugar 2,3-dehydratase | 98% | LobB1, AGI99483.1 |
| *ge00128* | Type I polyketide synthase | 98% | LobS4a, AGI99482.1 |
| *ge00129* | Type I polyketide synthase | 98% | LobS4b, AGI99482.1 |
| *ge00130* | Glycosyltransferase | 98% | LobA0, AGI99481.1 |
| *ge00131* | SAM-dependent methyltransferase | 99% | LobA9, AGI99480.1 |
| *ge00132* | Sugar 4-aminotransferase | 98% | LobA8, AGI99479.1 |
| *ge00133* | Sugar-O-methyltransferase | 99% | LobA7, AGI99478.1 |
| *ge00134* | Aldo/keto reductase | 99% | LobA6, AGI99477.1 |
| *ge00135* | p450 monooxygenase | 99% | LobA5, AGI99476.1 |
| *ge00136* | Efflux permease | 99% | LobA4, AGI99475.1 |
| *ge00137* | TetR type regulatory protein | 89% | LobR3, AGI99474.1 |
| *ge00138* | LobA3 | 100% | LobA3, AGC09508.1 |
| *ge00139* | LobA2 | 100% | LobA2, AGC09509.1 |
