# Supplementary material for: Multi-omics Data Reveal the Effect of Sodium Butyrate on Gene Expression and Protein Modification in Streptomyces
Source: Genomics Proteomics Bioinformatics. 2022 Sep 15;21(6):1149–62. doi: 10.1016/j.gpb.2022.09.002 (PMC11082262; doi:10.1016/j.gpb.2022.09.002)
Supplement: Supplementary Table S4 — 1H and 13C NMR assignment of lobophorin B (measured at 500 MHz in CDCl3) [file mmc10.docx]

**Table S4 ^1^H and ^13^C NMR assignment of lobophorin B (measured at 500 MHz in CDCl_3_)**

| **Number** | **^13^C** | **Δ ^1^H (multi., *J*)** |
| --- | --- | --- |
| 1 | 167.44 |  |
| 2 | 101.8 |  |
| 3 | 206.36 |  |
| 4 | 50.95 |  |
| 5 | 43.13 | 1.98 (t, 9.25) |
| 6 | 31.32 | 1.62 m |
| 7 | 41.7 | 1.50 (d, 9.0); 1.60 m |
| 8 | 34.43 | 2.22 m |
| 9 | 84.25 | 3.43 m |
| 10 | 38.43 | 2.08 m |
| 11 | 125.94 | 5.73 (d, 10.0) |
| 12 | 126.44 | 5.36 m |
| 13 | 53.2 | 3.44 m |
| 14 | 137.2 |  |
| 15 | 123.31 | 5.15 brs |
| 16 | 30.02 | 1.18 m, 2.33 m |
| 17 | 78.55 | 4.21 brs |
| 18 | 135.84 |  |
| 19 | 119.19 | 5.12 (d, 10.0) |
| 20 | 40.18 | 3.60 (d, 11.0) |
| 21 | 121.53 | 5.51 s |
| 22 | 141.42 |  |
| 23 | 27.93 | 2.68 (t, 7.2) |
| 24 | 35.39 | 1.85 (d, 14.5), 2.36 m |
| 25 | 83.46 |  |
| 26 | 201.67 |  |
| 27 | 15.08 | 1.61 s |
| 28 | 22.27 | 0.64 (d, 5.0) |
| 29 | 14.15 | 1.09 (d, 7.0) |
| 30 | 13.72 | 1.34 s |
| 31 | 15.11 | 1.40 s |
| 32 | 64.85 | 4.22 m |
| 33 | 20.18 | 1.32 (d, 7.5) |
| A-1 | 98.09 | 4.78 (d, 4.5) |
| A-2 | 31.04 | 1.68 m, 2.28 m |
| A-3 | 66.89 | 3.99 m |
| A-4 | 72 | 3.27 (dd, 3.5, 9.5) |
| A-5 | 64.97 | 3.99 m |
| A-6 | 18.27 | 1.26 (d, 5.5) |
| B-1 | 91.16 | 5.11 (d, 5.5) |
| B-2 | 34.23 | 1.90 （ddd，4.0，4.0，14.5）, 2.09 m |
| B-3 | 65.59 | 4.22 m |
| B-4 | 82.26 | 3.25 (dd, 3.0, 9.0) |
| B-5 | 62.12 | 4.00 m |
| B-6 | 17.71 | 1.21 (d, 6.0) |
| C-1 | 98.52 | 4.90 (dd, 2.0, 9.5) |
| C-2 | 36.79 | 1.63 m, 2.14 m |
| C-3 | 63.94 | 4.27 m |
| C-4 | 82.2 | 2.86 (dd, 3.0, 9.5) |
| C-5 | 68.41 | 3.77 m |
| C-6 | 17.9 | 1.25 (d, 6.0) |
| C-7 | 57.37 | 3.42 s |
| D-1 | 97.07 | 4.48 (dd, 2.0, 9.5) |
| D-2 | 35.61 | 1.60 m, 2.76 (d, 15.0) |
| D-3 | 91.04 |  |
| D-4 | 53.75 | 4.37 (d, 10.0) |
| D-5 | 69.06 | 3.50 m |
| D-6 | 16.97 | 1.16 (d, 6.0) |
| D-7 | 25.29 | 1.58 s |
| D-8 | 157.65 |  |
| D-9 | 52.77 | 3.72 s |
