# Supplementary material for: Multi-omics Data Reveal the Effect of Sodium Butyrate on Gene Expression and Protein Modification in Streptomyces
Source: Genomics Proteomics Bioinformatics. 2022 Sep 15;21(6):1149–62. doi: 10.1016/j.gpb.2022.09.002 (PMC11082262; doi:10.1016/j.gpb.2022.09.002)
Supplement: Supplementary Table S9 — Strains and plasmids used in this study [file mmc15.docx]

**Table S9 Strains and plasmids used in this study**

| **Strains/plasmids** | **Descriptions** | **References** |
| --- | --- | --- |
| **Strains** |  |  |
| JM109 | *F,* *proA^+^B^+^*, *lacI*^q^, Δ(*lacZ*)M15/Δ(*lac-proAB*), *gyrA96*, *recA1*, *relA1*, *endA1*, *hsdR17* | [1] |
| ET12567/pUZ8002 | *Dam, dcm, hsdS, Cm^R^, Str^R^, Tet^R^, Km^R^* | [2] |
| *S. olivaceus* FXJ 8.021 | Wild type strain | This study |
| Δ*lob* | A derivative strain of *S. olivaceus* FXJ 8.021, *ge00109* disruption mutant | This study |
| Δ*lobc* | △*lob* containing pSET152-109 | This study |
| *Baccillus cereus* CGMCC 1.1626 | Indicator strain for bioassays | This study |
| *Bacillus subtilis* CGMCC 1.1630 | Indicator strain for bioassays | This study |
| *Canidia albicans* CGMCC 2.4159 | Indicator strain for bioassays | This study |
| *Staphylococcus epidermidis* | Indicator strain for bioassays | This study |
| *Staphylococcus aureus* CGMCC 1.89 | Indicator strain for bioassays | This study |
| *Streptococcus pneumoniae* | Indicator strain for bioassays | This study |
| *Pseudomonas aeruginosa* | Indicator strain for bioassays | This study |
| **Plasmids** |  |  |
| pKC1139 | *aac*(*3*)*IV* (Apr^R^), temperature-sensitive plasmid | [1] |
| pKC1139-*lobUD* | pKC1139 containing two ~2.0 kb homologous fragments located upstream and downstream of *ge00109* | This study |
| pSET152-109 | pSET152 containing *ge00109* | This study |

*Note*: CGMCC, China General Microbiological Culture Collection Center; [1], Bierman M, Logan R, O'Brien K, Seno ET, Rao RN, Schoner BE. Plasmid cloning vectors for the conjugal transfer of DNA from *Escherichia coli* to *Streptomyces* spp. Gene 1992;116:43–9; [2], Paget MS, Chamberlin L, Atrih A, Foster SJ, Buttner MJ. Evidence that the extracytoplasmic function sigma factor *sigmaE* is required for normal cell wall structure in *Streptomyces coelicolor* A3(2). J Bacteriol 1999;181:204–11.
