# Supplementary material for: Multi-omics Data Reveal the Effect of Sodium Butyrate on Gene Expression and Protein Modification in Streptomyces
Source: Genomics Proteomics Bioinformatics. 2022 Sep 15;21(6):1149–62. doi: 10.1016/j.gpb.2022.09.002 (PMC11082262; doi:10.1016/j.gpb.2022.09.002)
Supplement: Supplementary Table S10 — Primers used in this study [file mmc16.docx]

**Table S10 Primers used in this study**

| **Primers** | **Sequences (5'***–***3')** |
| --- | --- |
| LobUp-F | GCTCTAGAAACCGGTACGAGGAAGGCCTC |
| LobUp-R | CCAAGCTTACGTCACATGGACCTGCGG |
| LobDn-F | CCAAGCTTTGTGGCGGCTGGTGGA |
| LobDn-R | CGGAATTCTCGAGGACGGGCCGTTCAC |
| Lobyz-F | GGACAAGCAGAACCGGCTG |
| Lobyz-R | ATGGCGAGATGCAACGCC |
| 00109-F1 | GCTCTAGACAGCAGTCCGGCGGCCGCT |
| 00109-R1 | GCGACATCGCCCCGACGAACA |
| 00109-F2 | AGGAGTACGGGCCGCGCCTCA |
| 00109-R2 | CGGAATTCTCACCAGGTGCCGGGCGACC |
| q-GE00109-F | TCAGTGAGTCGGAGGGCGTTC |
| q-GE00109-R | GATGCTCTCGCAGGCCAGGT |
| q-GE00100-F | CTGTTCGCATCTCGGTCGC |
| q-GE00100-R | CTCCTGCGTCAGCTGGTCG |
| q-GE07198-F | GAGGACAAGCTTCGCCAG |
| q-GE07198-R | GATGCCGGCACGTTCGAAC |
| q-GE00505-F | GTTGGTCAGCTGGAAGGCGAC |
| q-GE00505-R | CACCTACTTCCTGGCCACC |
| q-GE00211-F | TACCACCGACAACCGCACGC |
| q-GE00211-R | CTTCAGGGCCCAGTGGTACG |
| q-GE01054-F | ACGGCGACACACACCCCTCA |
| q-GE01054-R | CAGGTCGAACGGTGTCTCGCA |
| q16s-F | CGCTGGCGGCGTGCTTAA |
| q16s-R | GCTGGCACGTAGTTAGCC |
| C1-F | TCAGTGAGTCGGAGGGCGTTC |
| C1-R | GATGCTCTCGCAGGCCAGGT |
| C8-F | ACGGCGACACACACCCCTCA |
| C8-R | CAGGTCGAACGGTGTCTCGCA |
| C9-F | ATGGCGGGAAGGTTCCCG |
| C9-R | GATGGTGCCGTCGAAGCGC |
| C15-F | CTACCCGGAGTTCGACGAC |
| C15-R | GGCGTCAGGTTCCGCAGTA |
| C21-F | CAGTCCGGCATCTGGGTC |
| C21-R | CATGGTGGTAGCCGTGGAAG |
| C22-F | CAACGCGCGGATCCTGGA |
| C22-R | GTCCACCAGGTGCAGCTCG |
| C23-F | CGCCATCATCGGCATCGG |
| C23-R | GAGGCAGTGCGAGATGCGG |
| C26-F | AGCTCGACACGGAGAGCC |
| C26-R | GAAGCCGCTCTCCGGTAC |

*Note*: C1-F/R, C8-F/R, C9-F/R, C15-F/R, C21-F/R, C22-F/R, C23-F/R, and C26-F/R were used to amplify the DNA fragments of *ge00109* in C1, *ge01054* in C8, *ge01163* in C9, *ge03905* in C15, *ge05559* in C21, *ge05622* in C22, *ge05700* in C23, and *ge06020* in C26, respectively. C1, Cluster 1; C8, Cluster 8; C9, Cluster 9; C15, Cluster 15; C21, Cluster 21; C22, Cluster 22; C23, Cluster 23; C26, Cluster 26.
